# Gene Expression Signature of Cigarette Smoking and Its Role in Lung Adenocarcinoma Development and Survival

**DOI:** 10.1371/journal.pone.0001651

**Published:** 2008-02-20

**Authors:** Maria Teresa Landi, Tatiana Dracheva, Melissa Rotunno, Jonine D. Figueroa, Huaitian Liu, Abhijit Dasgupta, Felecia E. Mann, Junya Fukuoka, Megan Hames, Andrew W. Bergen, Sharon E. Murphy, Ping Yang, Angela C. Pesatori, Dario Consonni, Pier Alberto Bertazzi, Sholom Wacholder, Joanna H. Shih, Neil E. Caporaso, Jin Jen

**Affiliations:** 1 Division of Cancer Epidemiology and Genetics, National Cancer Institute, National Institutes of Health (NIH), Department of Health and Human Services (DHHS), Bethesda, Maryland, United States of America; 2 Center for Cancer Research, National Cancer Institute, National Institutes of Health (NIH), Department of Health and Human Services (DHHS), Bethesda, Maryland, United States of America; 3 Cancer Prevention Fellowship, National Cancer Institute, National Institutes of Health (NIH), Department of Health and Human Services (DHHS), Bethesda, Maryland, United States of America; 4 Division of Cancer Treatment and Diagnosis, National Cancer Institute, National Institutes of Health (NIH), Department of Health and Human Services (DHHS), Bethesda, Maryland, United States of America; 5 Biochemistry, Molecular Biology and Biophysics, University of Minnesota Cancer Center, Minneapolis, Minnesota, United States of America; 6 Department of Health Science, Mayo Clinic, Rochester, Minnesota, United States of America; 7 Foundation OM Policlinic, University of Milan, Milan, Italy; N, Minnesota State University Mankato, United States of America

## Abstract

**Background:**

Tobacco smoking is responsible for over 90% of lung cancer cases, and yet the precise molecular alterations induced by smoking in lung that develop into cancer and impact survival have remained obscure.

**Methodology/Principal Findings:**

We performed gene expression analysis using HG-U133A Affymetrix chips on 135 fresh frozen tissue samples of adenocarcinoma and paired noninvolved lung tissue from current, former and never smokers, with biochemically validated smoking information. ANOVA analysis adjusted for potential confounders, multiple testing procedure, Gene Set Enrichment Analysis, and GO-functional classification were conducted for gene selection. Results were confirmed in independent adenocarcinoma and non-tumor tissues from two studies. We identified a gene expression signature characteristic of smoking that includes cell cycle genes, particularly those involved in the mitotic spindle formation (e.g., NEK2, TTK, PRC1). Expression of these genes strongly differentiated both smokers from non-smokers in lung tumors and early stage tumor tissue from non-tumor tissue (p<0.001 and fold-change >1.5, for each comparison), consistent with an important role for this pathway in lung carcinogenesis induced by smoking. These changes persisted many years after smoking cessation. NEK2 (p<0.001) and TTK (p = 0.002) expression in the noninvolved lung tissue was also associated with a 3-fold increased risk of mortality from lung adenocarcinoma in smokers.

**Conclusions/Significance:**

Our work provides insight into the smoking-related mechanisms of lung neoplasia, and shows that the very mitotic genes known to be involved in cancer development are induced by smoking and affect survival. These genes are candidate targets for chemoprevention and treatment of lung cancer in smokers.

## Introduction

Lung cancer is the leading cause of cancer death worldwide. Cigarette smoking is responsible for about 90% of lung cancers and decreases survival,[1] and yet the precise molecular alterations induced by smoking in lung that develop into cancer and impact survival have remained obscure. Using Affymetrix HG-U133A microarrays on 135 fresh frozen adenocarcinoma and paired non-tumor tissue samples from current, former and never smokers from the Environment And Genetics in Lung cancer Etiology (EAGLE) study (http://dceg.cancer.gov/eagle), we sought to identify the genes that are altered by smoking in lung, and those, within the smoking signature, that have a role in lung carcinogenesis and outcome from lung cancer. We chose adenocarcinoma, the predominant histological subtype of lung cancer, because it occurs in subjects with no history of smoking as well as in smokers, providing a range of exposures ideal for the study of smoking-induced carcinogenesis. Specifically, in early stage adenocarcinoma tissue we compared gene expression from current (**C**) and never (**N**) smokers and identified the major genes using stringent criteria for gene selection (p<0.001 and fold change >1.5), the Benjamini-Hochberg procedure[2] to calculate the False Discovery Rate (FDR), and Gene Ontology (GO)[3] to classify the gene functional categories. We then verified whether the comparison between former (**F**) and never (**N**) smokers identified similar genes. We performed Gene Set Enrichment Analysis (GSEA)[4] to identify common gene patterns where the single-gene analysis revealed only few overlapping genes. We further explored whether the genes that differentiated lung tumors of smokers from never smokers (**C/N** and **F/N**) also differentiated early stage tumor tissue (**T**) from paired non-tumor (**NT**) tissue to confirm the role of these genes in smoking-related lung carcinogenesis. We finally explored the impact of the smoking signature on survival from lung cancer in smokers. We validated **C/N** genes by Real Time-PCR in 68 samples used for the present microarray analysis, and confirmed them in 40 independent samples from EAGLE and a Mayo Clinic study of lung cancer.

## Materials and Methods

### Study population and sample collection

This study included 105 subjects from EAGLE, a large population-based study of lung cancer conducted in the Lombardy region of Italy. EAGLE lung cancer cases were enrolled from the following 13 hospitals: A.O. Ospedale Niguarda Ca' Granda, Milano; A.O. Spedali Civili, Brescia; Istituto Clinico Humanitas, Rozzano (MI); Ospedale di Circolo e Fondazione Macchi, Varese; Fondazione IRCCS Ospedale Maggiore Policlinico, Mangiagalli and Regina Elena, Milano; Istituto Scientifico Universitario Ospedale San Raffaele, Milano; A.O. Ospedale Luigi Sacco, Milano; A.O. San Paolo, Milano; A.O. Ospedale San Carlo Borromeo, Milano; IRCCS Policlinico San Matteo, Pavia; A.O. San Gerardo, Monza; A.O. Ospedale Fatebenefratelli, Milano; Ospedale San Giuseppe, Milano. The healthy controls in EAGLE were randomly selected from the same residential area of the lung cancer cases. After description of the EAGLE study by the study personnel, and discussion with potential participants, written informed consent was obtained under a protocol approved by the Institutional Review Board of each participating hospital and by the National Cancer Institute (Bethesda, MD). Subjects in this gene expression study, 44–79 years old, had histologically confirmed primary adenocarcinoma of the lung, stages I–IV, and provided detailed smoking and medical history information.

Overall, 180 adenocarcinoma and non-tumor tissue samples were selected for the analyses, including duplicate or triplicate samples from 14 subjects for quality control. Samples had been snap-frozen in liquid nitrogen within 20 minutes of surgical resection. A single pathologist confirmed the hospital-based diagnosis of adenocarcinoma, estimated the presence of malignant cells in each sample based on H&E-stained fresh frozen sections, and classified the samples as Tumor (**T**) and Non-Tumor (**NT**). From the original 180 samples, 148 provided sufficient quantity of high-quality RNA for microarray analyses; 13 additional samples were excluded because of technical problems. Normalization was conducted on the remaining 135 microarrays; corresponding CEL files and information conform to the MIAME guidelines are publicly available on the GEO database (accession number = GSE10072). After normalization, 13 samples were excluded because of low percentage of tumor cells in the tumor tissues. This report is based on 122 samples, of which 15 duplicates/triplicates were averaged, resulting in 107 final expression values from 58 tumor and 49 non-tumor tissues from 20 never smokers, 26 former smokers, and 28 current smokers. Quality assurance and distribution of cell types across smoking groups are described in Appendix S1A, S1B, and S1C.

### Statistical analysis

All statistical analyses were accomplished using R program language. Gene expression data were processed and normalized using Bioconductor Affy package, based on the Robust Multichip Average (RMA) method[5] for single-channel Affymetrix chips. All 22,283 probe sets based on RMA summary measure were used in class comparison analyses.

Average linkage hierarchical clustering of samples was based on one minus Pearson correlation as the dissimilarity metric.

An ANOVA analysis adjusting for sex was used to test whether genes were differentially expressed between smoking groups (**C/N** and **F/N**), between tumor tissue and non-tumor tissue (**T/NT**), or by pack years of cigarette smoking. Further analyses adjusted by tumor grade or excluding 6 subjects with emphysema or chronic bronchitis or 3 subjects who received chemotherapy prior to the study were conducted, with essentially unaltered results. For analyses including paired tissues (**T/NT** tissue samples from the same subjects), a linear mixed effects model was used to account for intra-person correlation.

To limit false positive findings, genes were considered statistically significant if their p-values were less than the stringent threshold of 0.001. Under the null hypothesis of no difference in expression profiles, and considering the analysis of 22,283 probes, we expect that by chance the average number of false positive findings will be ≤23. We used the Benjamini-Hochberg[2] procedure to calculate the False Discovery Rate (FDR). We further restricted significant genes to those which showed at least 1.5 fold ratio of geometric means of expression between two groups. Gene selection based on p<0.001 (two-sided) and fold-change >1.5 are referred to as “stringent criteria”.

The Cox Proportional Hazards model[6] was used to estimate the effect of gene expression changes in **C/N** on survival from lung cancer in smokers. Of the 74 subjects included in this study (all stages), 34 (22 smokers) were alive, and 40 (32 smokers) were deceased as of May 2007. Among the deceased subjects, 36 died of lung cancer. The remaining 4 (2 smokers) died of other cancers and were censored at time of death in the analysis. The time from lung cancer to death or date of last follow-up was between 28 days and 5.0 years for the deceased subjects, and 3.7 and 5.7 years for the subjects alive in May 2007. The relative risk of gene expression was defined as the hazard ratio associated with one standard deviation change of the expression. Analyses were adjusted for stage, sex, and smoking. Age was similarly distributed across the groups and was not adjusted for in the analysis.

### Analysis of total plasma cotinine concentration by gas chromatography/mass spectrometry

We verified the self-reported current smoking status by measuring plasma cotinine levels. The total cotinine (free plus cotinine *N*-glucuronide) concentration in plasma was quantified by GC/MS analysis using a method similar to that used for urinary cotinine,[7] with the addition of a solid phase extraction step carried out on an MCX column (Waters Corporation, Milford, MA).

One individual who reported to have quit smoking 2.6 years before the study had high cotinine levels (135 ng/ml) and was re-classified as a current smoker.

### Gene Set Enrichment Analysis

Gene Set Enrichment Analysis (GSEA)[4] was used to compare expression in groups of genes (gene-sets), between different tissues or between different comparison groups within the same tissue. GSEA analysis reveals a pattern of common gene-sets even when single-gene analysis reveals very few overlapping genes between groups. We modified the standard GSEA method by substituting an ANOVA test for the standard two-sample t-test to adjust for sex. Furthermore, we changed the permutation test for calculating the p-values by permuting residuals and using as weights the observed ANOVA coefficients divided by the standard error values. Up- and down-regulated genes were included in different gene-sets for the analyses.

### Molecular function classification of smoking-altered genes

Gene Ontology was used to assign the genes to functional categories.[3] GoMiner[8] was utilized to rank-order the GO categories for the genes identified in the smoking comparisons.

### Quantitative PCR validation and confirmation in independent samples

We used quantitative real-time PCR (QRTPCR) to confirm the differential expression of 19 **C/N** selected genes (20 probes), including 14 genes from **T** and 5 from **NT** analyses. Primer and probe sets for the selected genes as well as control probes for GUSB and S18 (ABI) were run on 7500 Taqman under the manufacturer's standard protocol. Ct values were normalized based on GUSB expression.

Validation assays were performed in 68 samples used in the original microarray analyses, including 43 **T** (27 **C** and 16 **N** smokers), and 25 **NT** (18 **C** and 7 **N** smokers).

Confirmation assays were performed in 40 independent samples, including 19 **T** (12 **C** and 7 **N** smokers) and 21 **NT** samples (12 **C** and 9 **N** smokers). These samples were collected in EAGLE (10 **T** samples from 7 **C** and 3 **N** smokers, and 12 **NT** samples from 7 **C** and 5 **N** smokers-these samples were not used for the microarray analyses), and from the Mayo Clinic, Rochester, MN (9 **T** and 9 **NT** paired samples from 5 **C** and 4 **N** smokers).

## Results

### The molecular signature of cigarette smoking in lung adenocarcinoma

To investigate the molecular changes associated with smoking in the tumor tissue, we compared gene expression changes between current and never (**C/N**) smokers (Table 1). To avoid potential alteration of gene expression due to advanced tumor status, we limited smoking comparisons in tumor tissue to the early stages (stages I and II). Unless specified differently, “**T**” samples represent early stage adenocarcinomas. Results from the advanced tumor stage tissues are reported for completeness in Appendix S2C.

**Table 1:** Number of probes and genes differentiating current from never smokers (C/N) and former from never smokers (F/N) in all tumor samples, early stage tumor samples (T), and all non-tumor (NT) tissue samples.

Criteria for significance		All stages Tumor				Stages I and II Tumor (“T”)				Non-Tumor (“NT”)			
	Comparison between smokers	24 Current v*s.* 16 Never		18 Former v*s.* 16 Never		20 Current *vs.* 10 Never		13 Former *vs.* 10 Never		16 Current *vs.* 15 Never		18 Former *vs.* 15 Never	
	FDR^a^	8.5%		17.0%		9.5%		27.8%		7.8%		78.3%	
	Direction	Down	Up	Down	Up	Down	Up	Down	Up	Down	Up	Down	Up
**p-value<0.001**	**Probes**	142	119	25	105	126	106	31	40	211	71	7	2
	**Genes**	119	104	22	97	104	89	25	35	191	64	7	2
**p-value<0.001+Fold change>1.5**	**Probes**	61	63	17	3	98	64	26	4	75	28	1	0
	**Genes**	48	56	15	3	81	54	21	4	73	25	1	0

a FDR = False Discovery Rate [2]

Using stringent selection criteria (fold-change >1.5 and p-value<0.001), we identified 64 up- and 98 down-regulated probe-sets, representing 54 up- and 81 down-regulated genes (Appendix S2A, S2B). Most of the significantly up-regulated genes were involved in cell cycle/mitosis/cell division (e.g., TTK, CENPF, NEK2), while many of those down-regulated were involved in cell adhesion/cell cycle arrest (e.g., ADRB2, APLP2, MACF1), consistent with a role of these genes in neoplasia development.

The GoMiner results (Appendix S2D) confirmed that the mitosis genes (12 altered genes among the 127 mitotic genes on the HG-U133A chip, p<0.001), and more generally those involved in cell cycle were the most commonly altered in the tumor tissue (Table 2).

**Table 2:** Cell cycle genes differentiating current from never smokers (C/N) in the early stage tumor (T) tissue samples, and corresponding values in the former/never smoker (F/N) and in the smokers' paired tumor/non-tumor tissue (T/NT) comparisons.

Probe ID	Gene	Chromosomal	Current/Never† N = 30		Former/Never N = 23		Tumor/Non-Tumor N = 36	
	Symbol	Location	Fold-change	p-value	Fold-change	p-value	Fold-change	p-value
204641_at	NEK2*	1q32.2–q41	3.45	0.0001	2.84	0.0036	3.14	<0.0001
204822_at	TTK*	6q13–q21	3.27	<0.0001	2.08	0.0123	2.22	<0.0001
218009_s_at	PRC1*	15q26.1	2.99	0.0007	2.61	0.0109	2.60	<0.0001
207828_s_at	CENPF*	1q32–q41	2.88	<0.0001	2.28	0.0034	2.77	<0.0001
202095_s_at	BIRC5*	17q25	2.72	0.0002	2.10	0.0145	2.55	<0.0001
203362_s_at	MAD2L1	4q27	2.67	0.0003	1.93	0.0309	2.74	<0.0001
219918_s_at	ASPM	1q31	2.59	0.0008	2.12	0.0218	2.87	<0.0001
210559_s_at	CDC2	10q21.1	2.54	0.0009	2.02	0.0298	2.37	<0.0001
201897_s_at	CKS1B	1q21.2	2.36	0.0002	1.89	0.0152	2.47	<0.0001
204170_s_at	CKS2	9q22	2.36	0.0006	2.02	0.0148	1.69	0.0015
222077_s_at	RACGAP1*	12q13.12	2.35	0.0003	1.91	0.0178	2.13	<0.0001
203214_x_at	CDC2	10q21.1	2.29	0.0006	1.98	0.0150	2.12	<0.0001
219306_at	KIF15*	3p21.31	2.22	0.0002	2.00	0.0047	1.90	0·0001
209642_at	BUB1*	2q14	2.17	0.0009	1.68	0.0507	2.02	0.0001
210052_s_at	TPX2*	20q11.2	2.06	0.0006	1.87	0.0100	2.07	<0.0001
203418_at	CCNA2	4q25–q31	1.99	<0.0001	1.85	0.0012	1.82	<0.0001
212020_s_at	MKI67	10q25-qter	1.95	<0.0001	1.71	0.0016	1.41	0.0006
201088_at	KPNA2	17q23.1–q23.3	1.82	<0.0001	1.53	0.0079	2.34	<0.0001
211519_s_at	KIF2C*	1p34.1	1.78	0.0004	1.67	0.0062	1.51	0.0002
218252_at	CKAP2	13q14	1.75	0.0008	1.52	0.0292	1.47	0.0001
204887_s_at	PLK4	4q27–q28	1.74	0.0001	1.55	0.0066	1.48	<0.0001
211080_s_at	NEK2*	1q32.2–q41	1.57	0.0001	1.50	0.0019	1.36	0.0002
214894_x_at	MACF1	1p32–p31	0.65	0.0003	0.64	0.0016	0.52	<0.0001
208634_s_at	MACF1	1p32–p31	0.60	0.0001	0.58	0.0004	0.42	<.0.0001
202284_s_at	CDKN1A	6p21.2	0.54	0.0003	0.70	0.0668	0.65	0.0082
208893_s_at	DUSP6	12q22–q23	0.34	0.0003	0.32	0.0012	0.84	0.3102

† Probe selection restricted to estimates with p<0.001 and fold-change >1.5 or <0.6667, and within the most inclusive category of genes with p≤0.001 in the GoMiner analysis (GO ID 7049, Appendix S2D).

* Genes involved in the mitotic spindle formation. The double line separates up-regulated and down-regulated probes.

### Lung cancer gene expression is similar in current and former smokers

**Figure 1:**
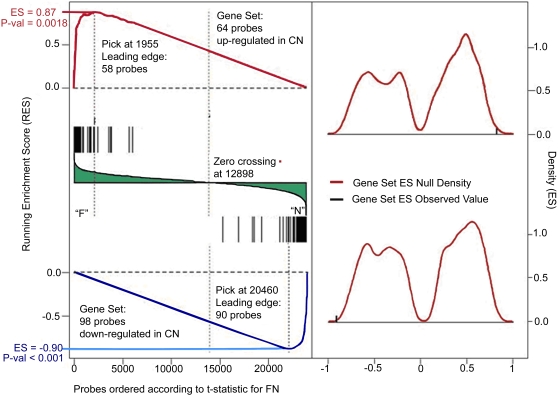
Comparison of gene expression differentiating current from never smokers (C/N) and gene expression differentiating former from never smokers (F/N) in early stage tumor tissue (T) using Gene Set Enrichment Analysis (GSEA). Left: Running Enrichment Score (y axis) is calculated by walking down the entire list of probes from Affymetrix HG-U133A chip (numbered from 1 to 22,283 in the x axis) ordered by the ANOVA coefficients divided by the standard error values from the Former/Never (F/N) smoking comparison. This running-sum statistic increases when a given probe is in the Current/Never (C/N) Gene Set of interest and decreases when the probe is not in the C/N Gene Set, with the magnitude of increment depending on the strength of the correlation between the probe and the F/N comparison. The Enrichment Score (ES) is the maximum deviation of the Running Enrichment Score from zero encountered in the random walk and reflects the degree to which the Gene Set is overrepresented at the extremes (top or bottom) of the entire ranked probe list. We report results for two different C/N Gene Sets: on the top, the 64 up-regulated probes, with ES = 0.87 and, on the bottom, the 98 down-regulated probes, with ES = −0.90. A leading edge subset of the Gene Set is defined as those probes in the Gene Set that appear in the probes ranked list at, or before, the point where the running sum reaches its maximum deviation from zero. The leading edge for the Gene Set of the C/N up-regulated probes contains 58 probes over 64 and the leading edge for the Gene Set of down-regulated probes contains 90 over 98 probes. This confirms that among the 64 up-regulated probes from the C/N comparison, 58 are also found in the F/N comparison; and among the 98 down-regulated probes from the C/N comparison, 90 are also found in the F/N comparison. Right: distributions of ES values created using a permutation procedure for (top) the Gene Set of up-regulated probes in C/N and (bottom) the Gene Set of down-regulated probes in C/N. These distributions are used to calculate the statistical significance (nominal p-value) of the observed ES values (p-value<0.002 in both cases).

To verify whether the **C/N** smoking signature in the tumor was present also in former smokers, we compared the **C/N** and **F/N** signatures in **T** and found 26 probes (22 down- and 4 up-regulated, representing 21 genes) that differentiated both **C/N** and **F/N** using stringent selection criteria (Appendix S2E). Some of these genes, e.g., STOM, SSX2IP, TRPC6, APLP2 (2 probes), and DHRS7, exhibited a persistent alteration even in subjects (n = 6) who quit smoking more than 20 years before the study. The GSEA analysis showed that among the 64 up- and 98 down-regulated probes found in the **C/N** comparison in **T**, 58 and 90 probes, representing 50 up- and 73 down-regulated genes, were also up- and down-regulated, respectively in the **F/N** smoking comparison (p<0.001, Fig. 1, and Appendix S2F, S2G). All cell cycle genes that differentiated **C/N** were also altered in **F/N**, although less prominently (Table 2), indicating that alterations of these genes persist following smoking cessation. Importantly, the mitosis/cell cycle genes identified in **C/N** and **F/N** also differentiated the early stage tumor from the non-tumor tissue samples (**T/NT,** paired analysis) (Table 2), while pack years of cigarette smoking, a composite index of intensity and duration that does not consider the time when smoking occurred, were not associated with gene expression in either **T** or **NT**.

### Smoking signature in the noninvolved lung tissue

The **C/N** comparison in **NT** revealed 28 up- and 75 down-regulated probes, representing 25 up- and 73 down-regulated genes with the stringent selection criteria (Table 1, and Appendix S3A, S3B). As expected, the CYP1B1 gene, known to be induced by smoking[9], [10] was strongly up-regulated. The GoMiner results showed that the most smoking-altered genes were involved in cellular defense response (5 of 90 cellular defense genes on the chip, p<0.001), and more generally in immune response (Appendix S3C).

MACF1, UBE21, and CBX7 (p<0.001), and C16orf30 (p = 0.001) were shared between **T** and **NT** **C/N** comparisons. C16orf30 and UBE21, both on chromosome 16p13.3, are located within 246kb, but they do not appear to share specific transcriptional regulation mechanisms (Appendix S4A). The GSEA analysis revealed some similarities between **T** and **NT** in the overall pattern of smoking-induced alteration (p = 0.08 and 0.04, for up- and down-regulated genes, respectively, Appendix S4B, S4C, and S4D). Notably, NEK2 and TTK were among those similarly altered in both **T** and **NT** in the GSEA analysis. In contrast, the **F/N** comparison in **NT** showed no statistically significant genes (Table 1), and was not further explored.

### Smoking-associated gene expression signature and survival from lung cancer

We studied the overall gene expression signature of smoking in **T** and **NT** (98+64 **C/N** in **T**, 75+28 **C/N** in **NT**, minus 3 overlapping probes between **T** and **NT**, for a total of 262 probe-sets representing 230 genes) in relation to survival from adenocarcinoma in smokers (n = 54, Appendix S5A). Since only 262 probe-sets were included in this analysis, we used a less stringent criterion of p<0.01 for gene selection (Table 3). Altered expression in **NT** of genes involved in the mitotic spindle formation, e.g., NEK2 (p<0.001) and TTK (p = 0.001) were associated with a 3-fold increased mortality risk (Table 3, analysis adjusted for stage, sex, and smoking).

**Table 3:** Mortality risk in smokers for gene expression differentiating current from never smokers (C/N) in lung tumor and non-tumor tissue samples (p<0.01)

Probe ID	Gene Symbol	p-value	RR^a^	95% CI^b^ Lowest	95% CI^b^ Highest	Tissue type^c^
**Increased risk**						
204641_at	NEK2	0.0008	2.6	1.5	4.7	NT
204822_at	TTK	0.0011	2.9	1.5	5.5	NT
201292_at	TOP2A	0.0041	3.1	1.4	6.7	NT
219306_at	KIF15	0.0048	2.8	1.4	5.9	NT
218542_at	C10orf3	0.0068	2.7	1.3	5.4	NT
209642_at	BUB1	0.0084	2.8	1.3	5.9	NT
201637_s_at	FXR1	0.0007	2.8	1.5	5.0	T
213189_at	DKFZp667G2110	0.0088	2.0	1.2	3.4	T
**Decreased risk**						
202068_s_at	LDLR	0.0068	0.5	0.3	0.8	NT
214894_x_at	MACF1	0.0091	0.4	0.2	0.8	NT
218804_at	TMEM16A	0.0095	0.4	0.2	0.8	NT
201651_s_at	PACSIN2	0.0046	0.4	0.2	0.8	T

a Relative Risk of death. Analysis based on 54 current and former smokers using 262 probes from the Current/Never smoking comparisons (98 down- and 64 up-regulated probes from T and 75 down- and 28 up-regulated probes from NT, minus 3 overlapping probes in T and NT); models adjusted for tumor stage, sex, and smoking status

b 95% Confidence Interval

c T = Lung adenocarcinoma samples of any stage (N = 42); NT = Non-Tumor tissue samples (N = 34)

### Validation and confirmation of gene expression smoking signature

We selected 19 genes (20 probes) for validation by QRTPCR, including 14 genes for **T** and 5 for **NT** tissue, based on fold change (>2) and cancer relevance.

Validation was based on 68 samples, including 43 **T** and 25 **NT**, also used for the microarray analysis. All 19 genes were up-regulated in the **C/N** comparison in these samples (Table 4).

**Table 4:** Quantitative Real-Time PCR validation of microarray results of genes differentiating current from never smokers (C/N) in tumor (T) and non-tumor (NT) tissue samples and confirmation in independent samples

Gene Name	ABI Assay ID	Average expression in validation samples^a^			Average expression in confirmation samples^b^		
		Current Smokers	Never Smokers	Fold Change C/N^c^	Current Smokers	Never Smokers	Fold Change C/N^c^
Early Stage Adenocarcinoma		N = 27	N = 16		N = 12	N = 7	
AURKA	Hs00269212_m1	16.57	17.37	1.74	19.13	20.21	2.10
BIRC5	Hs00153353_m1	10.48	11.46	1.97	12.31	13.42	2.16
BIRC5	Hs00977611_g1	10.84	12.45	3.07	13.13	14.24	2.16
CCNA2	Hs00153138_m1	13.13	13.42	1.22	15.16	15.93	1.71
CENPF	Hs00193201_m1	11.23	12.87	3.13	12.89	13.00	1.08
C10orf23	Hs00216688_m1	11.78	12.58	1.74	13.72	14.74	2.03
CKS1B	Hs01029137_g1	9.36	9.91	1.46	11.19	11.69	1.41
FOXM1	Hs00153543_m1	10.43	11.65	2.33	12.25	12.92	1.60
GGH	Hs00608257_m1	11.92	13.75	3.55	14.79	15.70	1.88
KIF20A	Hs00194882_m1	12.40	13.65	2.38	15.26	15.76	1.41
KIF4A	Hs00602211_g1	12.42	13.66	2.37	14.64	15.82	2.27
MKI67	Hs00267195_m1	12.53	12.71	1.14	13.95	14.57	1.54
NEK2	Hs00601227_mH	12.61	14.52	3.74	15.91	18.01	4.27
TPX2	Hs00201616_m1	10.60	11.73	2.19	12.57	13.30	1.66
TTK	Hs00177412_m1	11.49	12.80	2.47	13.12	13.28	1.11
**Non-Tumor Lung Tissue**		**N = 18**	**N = 7**		**N = 12**	**N = 9**	
CEACAM5	Hs00237075_m1	10.98	14.85	14.58	13.30	13.51	1.16
CYBB	Hs00166163_m1	5.86	8.19	5.03	8.20	9.30	2.15
CYTL1	Hs00184064_m1	11.54	14.61	8.36	14.16	14.09	0.96
FGG	Hs00241037_m1	9.05	11.99	7.71	11.03	15.36	20.13
TM7SF4	Hs00229255_m1	14.99	18.20	8.15	17.42	18.47	2.07

a Validation in 68 samples used also for microarray analysis;

b Confirmation in 40 independent samples from EAGLE and Mayo Clinic

c C/N = Current/Never smoking comparison

Confirmation was based on 40 independent samples (19 **T** and 21 **NT**) from EAGLE (samples not used for microarray analysis) and the Mayo Clinic, Rochester, MN. All the 14 genes in **T** and 4 of 5 genes in **NT** were up-regulated by smoking also in the independent samples (Table 4).

## Discussion

In a population-based study with fresh frozen tissue samples of adenocarcinoma and noninvolved lung tissue (mostly paired samples), we identified a smoking signature that persists years after smoking cessation and is related to lung cancer development and survival.

Aneuploidy and chromosome instability are two of the most common abnormalities in cancer cells that arise through unequal segregation of chromosomes between daughter cells during mitosis. Thus, mitotic alterations are highly relevant for carcinogenesis. We found that smoking induces deregulation of this very mitotic process proceeding from lung tissue changes through cancer development to cancer death or survival. In fact, the smoking signature we identified comprises genes that regulate the mitotic spindle formation. These genes, such as NEK2[11], [12] and CENPF[11] (both on 1q32-q41), TPX2[13], [14] and STK6 (or AURKA)[15] (related to the Aurora-A activation pathway important in tumor progression[16]), TTK (linked to cell mitosis through EGFR,[17] a critical drug target for lung adenocarcinoma[18]), and BIRC5 (Survivin),[19] have all been found over-expressed in smoking-related tumors. While previous studies have proposed these genes as targets for therapeutic interventions,[16], [18]–[21] our work suggests that they may be targets for chemoprevention in smokers as well. In fact, they were strongly induced by smoking in the early stage tumor tissue and some, e.g., NEK2 and TTK, were also associated with increased mortality risk. The latter finding was most evident in non-tumor tissue, likely reflecting the widely recognized field-cancerization effect by smoking,[22] while in the tumor tissue, smoking-related genes' effects on survival may be masked by extensive molecular alterations occurring during tumorigenesis.

In the non-tumor tissue, current smoking strongly altered immune response genes, consistent with the defense mechanisms of the lung tissue against the acute toxic effects of smoking. Among the gene most strongly down-regulated in **NT** was CX3CR1, located on chromosome 3p21.3, an area known to be often deleted in lung cancer,[23] particularly in smokers.[24]

Current knowledge of gene expression altered by cigarette smoking is based on bronchoscopy-obtained airway epithelial cells or macrophages[9], [25]–[27] or peripheral leukocytes[10] from healthy smokers rather than directly on lung tissue. The few studies with lung tissue samples are very small[28] or used RNA amplification[29] or RNA pooling[30] methods. Our results are consistent with some previous findings, such as smoking-related alteration of CYP1B1[9], [10] or of the mitotic pathway in cancer survival.[29] However, earlier studies were often limited by the small sample size, or lacked information on potential confounders, or availability of paired tumor and non-tumor lung tissue samples for the distinction of gene changes involved in lung carcinogenesis from those representing a transient smoking effect. We overcame these pitfalls with a relatively large sample size of fresh tumor and non-tumor lung tissues, detailed covariate information (e.g., sex, age, stage, previous lung diseases or chemotherapy), biochemical validation of the smoking status, and confirmation of the main findings in independent tissue samples.

In conclusion, our study provides clues on how cigarette smoking affects lung cancer development and survival. Functional assays to confirm these findings are warranted. If confirmed, these genes could become important targets for chemoprevention and treatment for lung cancer in smokers.

## Supporting Information

Appendix S1 Quality Assurance. 1A Description of analysis of sample quality assurance 1B Samples' description 1C Surfactant genes in Tumor (T) and Non-Tumor (NT) lung tissues by smoking
(0.07 MB DOC)

Appendix S2 Current/Never (C/N) and Former/Never (F/N) smoking comparisons in early stage Tumor (T) tissue. 2A Current/Never (C/N) comparison, early stage Tumor (T) tissues: up-regulated probes. 2B Current/Never (C/N) comparison, early stage Tumor (T) tissues: down-regulated probes. 2C Current/Never (C/N) comparison, late stage Tumor tissues: up+down-regulated probes. 2D Gene Ontology (GO) functional categories for the Current/Never (C/N) smoker comparison. 2E Current/Never (C/N) and Former/Never (F/N) comparisons: overlapping probe list. 2F Gene list from GSEA comparison of up-regulated C/N genes and F/N genes in early stage Tumor (T) tissues. 2G Gene list from GSEA comparison of down-regulated C/N genes and F/N genes in early stage Tumor (T) tissues.
(0.62 MB DOC)

Appendix S3 Current/Never (C/N) smoking comparisons in Non-Tumor (NT) lung tissue. 3A Current/Never (C/N) comparison in Non-Tumor (NT) lung tissues: up-regulated probes. 3B Current/Never (C/N) comparison in Non-Tumor (NT) lung tissues: down-regulated probes . 3C Gene Ontology (GO) functional categories for the Current/Never (C/N) comparison (up and down-regulated genes) in Non-Tumor (NT) lung tissues.
(0.21 MB DOC)

Appendix S4 Comparison between Tumor (T) and Non-Tumor (NT) lung tissue for the genes whose expression significantly differentiates Current from Never smokers (C/N) in early stage lung Tumor (T). 4A C16orf30 and UBE21 transcription sites. 4B Comparison of C/N results in early stage Tumor (T) tissues vs. C/N results in Non-Tumor (NT) lung tissues by GSEA analysis. 4C Gene list from GSEA comparison of up-regulated C/N genes between early stage Tumor (T) tissues and Non-Tumor (NT) tissues. 4D Gene list from GSEA comparison of down-regulated C/N genes between early stage Tumor (T) tissues and Non-Tumor (NT) tissues.
(0.51 MB DOC)

Appendix S5 Mortality risk in smokers associated with the expression of genes differentiating Current from Never smokers (C/N) in Tumor and Non-Tumor tissue samples. 5A Current/Never (C/N) genes and related mortality risk in Tumor and Non-Tumor lung tissues (all stages) from Current and Former smokers.
(0.55 MB DOC)
