# Supplementary material for: Gene Expression Signature of Cigarette Smoking and Its Role in Lung Adenocarcinoma Development and Survival
Source: PLoS One. 2008 Feb 20;3(2):e1651. doi: 10.1371/journal.pone.0001651 (PMC2249927; doi:10.1371/journal.pone.0001651)
Supplement: Appendix S1 — Quality Assurance. 1A Description of analysis of sample quality assurance 1B Samples' description 1C Surfactant genes in Tumor (T) and Non-Tumor (NT) lung tissues by smoking (0.07 MB DOC) [file pone.0001651.s001.doc]

**Appendix S1**

**Quality assurance**

**Supplementary Text 1A**

**Description of analysis of sample quality assurance**

A single pathologist ascertained the presence or absence of tumor cells in each sample, and confirmed the original histology-based diagnosis of adenocarcinoma. Sample description by smoking groups is shown in Supplementary Table 1B. To verify the final sample classification, we compared gene expression in tumor tissue and noninvolved tissue samples from the same subjects (early stage tumors, T). As expected, we observed distinct clustering by tissue type within each smoking group. Overall, there were 2436 probes (1920 genes) that significantly (p<0.001) distinguished T from NT. Among the 1066 genes with a fold change >1.5 and p<0.001, 116 were similarly altered in each smoking group, while 261, 364, and 60 genes differentiated T/NT tissue only in current smokers, former smokers, and never smokers, respectively. The remaining 265 genes were similarly altered in some but not all smoking groups. The gene list from the Tumor vs. Non-Tumor comparison is available upon request.

We opted not to conduct microdissection of tumor samples to avoid the necessity for RNA amplification or pooling. To minimize potential gene expression differences due to tissue heterogeneity across smoking groups, we verified that the samples from the left and right lungs and upper and lower lobes were similarly distributed across smoking groups, and the average intensity of the genes differentiating the current from never (C/N) smokers was similar by tumor anatomical site. Moreover, the average expression intensity of the genes typically expressed in the distant alveolar epithelium (e.g., surfactant SFTPA2, SFTPC (3 probes), SFTPD)[1] were similarly expressed in the tumor and noninvolved tissue samples across the different smoking groups (results in Supplementary Table 1C), supporting the hypothesis that gene expression differences between C/N smokers were not due to differential alveolar cell types in the samples. We also verified that the list of selected genes differentiating C/N smoking in the tumor and noninvolved lung tissue samples did not largely include those from inflammatory or white blood cells (e.g., B cells’ CD79, T cells’ CD3, monocytes’ MPO, myeloid specific cells’ CD33, CD11c, or CD61[1], or macrophages’ CSF1[2]).

Reference List

1. Spira A, Beane J, Shah V, Liu G, Schembri F, Yang X, Palma J, Brody JS (2004) Effects of cigarette smoke on the human airway epithelial cell transcriptome. Proc Natl Acad Sci U S A 101: 10143-10148.

2. Heguy A, O'Connor TP, Luettich K, Worgall S, Cieciuch A, Harvey BG, Hackett NR, Crystal RG (2006) Gene expression profiling of human alveolar macrophages of phenotypically normal smokers and nonsmokers reveals a previously unrecognized subset of genes modulated by cigarette smoking. J Mol Med 84: 318-328.

**Supplementary Table 1B**

**Samples’ description**

| **Smoking** | **All Tumor** | **All Non-Tumor** | **Tumor Stages I+II** | **Tumor Stages III+IV** | **All Paired T + NT** | **Paired T + NT Stages I+II** | **Paired T + NT Stages III + IV** | **Lung cancer deaths**  **Tumor** | **Lung cancer deaths**  **Non-Tumor** | **Living as of May 2007**  **Tumor** | **Living as of May 2007**  **Non-Tumor** | **Other causes of death**  **T+NT** |
| --- | --- | --- | --- | --- | --- | --- | --- | --- | --- | --- | --- | --- |
| **Never** | 16 | 15 | 10 | 6 | 11 | 6 | 5 | 6 | 4 | 9 | 9 | 3 |
| **Former** | 18 | 18 | 13 | 5 | 10 | 9 | 1 | 12 | 14 | 6 | 4 | 0 |
| **Current** | 24 | 16 | 20 | 4 | 12 | 9 | 3 | 11 | 5 | 11 | 10 | 3 |
| **Total** | 58 | 49 | 43 | 15 | 33 | 24 | 9 | 29 | 23 | 26 | 23 | 6 |

**Supplementary Table 1C**

**Surfactant gene expression in Tumor (T) and Non-Tumor (NT) lung tissue by smoking.**

| **Early Stage Tumor Tissue** |  |  |  |  |  |  |  |  |
| --- | --- | --- | --- | --- | --- | --- | --- | --- |
| Probe ID | Gene Symbol | Average Intensity Current | Average Intensity Former | Average Intensity Never | Fold-change C/N | Fold-change F/N | p-value C/N | p-value F/N |
| 205982_x_at | SFTPC | 11.18 | 11.26 | 11.79 | 0.43 | 0.37 | 0.18 | 0.19 |
| 211735_x_at | SFTPC | 11.25 | 11.40 | 11.95 | 0.40 | 0.34 | 0.16 | 0.16 |
| 214199_at | SFTPD | 10.38 | 10.02 | 10.67 | 0.61 | 0.48 | 0.26 | 0.16 |
| 215454_x_at | SFTPC | 7.25 | 7.33 | 7.79 | 0.54 | 0.49 | 0.13 | 0.15 |
| 218835_at | SFTPA2 | 12.83 | 12.81 | 13.78 | 0.26 | 0.32 | 0.03 | 0.10 |
|  |  |  |  |  |  |  |  |  |
| **Noninvolved Lung Tissue** |  |  |  |  |  |  |  |  |
| Probe ID | Gene Symbol | Average Intensity Current | Average Intensity Former | Average Intensity Never | Fold-change C/N | Fold-change F/N | p-value C/N | p-value F/N |
| 205982_x_at | SFTPC | 14.45 | 14.41 | 14.61 | 0.90 | 0.78 | 0.61 | 0.29 |
| 211735_x_at | SFTPC | 14.51 | 14.51 | 14.65 | 0.91 | 0.80 | 0.65 | 0.35 |
| 214199_at | SFTPD | 12.71 | 12.60 | 12.78 | 0.97 | 0.83 | 0.89 | 0.44 |
| 215454_x_at | SFTPC | 11.05 | 11.05 | 11.20 | 0.90 | 0.81 | 0.67 | 0.46 |
| 218835_at | SFTPA2 | 14.40 | 14.29 | 14.38 | 1.01 | 0.85 | 0.95 | 0.39 |

C/N= Current/Never smoking comparison

F/N= Former/Never smoking comparison
