# Supplementary material for: Gene Expression Signature of Cigarette Smoking and Its Role in Lung Adenocarcinoma Development and Survival
Source: PLoS One. 2008 Feb 20;3(2):e1651. doi: 10.1371/journal.pone.0001651 (PMC2249927; doi:10.1371/journal.pone.0001651)
Supplement: Appendix S2 — Current/Never (C/N) and Former/Never (F/N) smoking comparisons in early stage Tumor (T) tissue. 2A Current/Never (C/N) comparison, early stage Tumor (T) tissues: up-regulated probes. 2B Current/Never (C/N) comparison, early stage Tumor (T) tissues: down-regulated probes. 2C Current/Never (C/N) comparison, late stage Tumor tissues: up+down-regulated probes. 2D Gene Ontology (GO) functional categories for the Current/Never (C/N) smoker comparison. 2E Current/Never (C/N) and Former/Never (F/N) comparisons: overlapping probe list. 2F Gene list from GSEA comparison of up-regulated C/N genes and F/N genes in early stage Tumor (T) tissues. 2G Gene list from GSEA comparison of down-regulated C/N genes and F/N genes in early stage Tumor (T) tissues. (0.62 MB DOC) [file pone.0001651.s002.doc]

**Appendix S2**

**Current/Never (C/N) and Former/Never (F/N) smoking comparisons in**

**early stage Tumor (T) tissues**

**Supplementary Table 2A**

**Current/Never (CN) comparison, early stage Tumor (T) tissues: up-regulated probes**

| **Probe ID** | **CN**  **Fold Change >1.5** | **CN**  **p-value < 0.001** | **Gene Symbol** | **Chromosomal Location** |
| --- | --- | --- | --- | --- |
| 201291_s_at | 3.5307 | 0.0002 | TOP2A | 17q21-q22 |
| 204641_at | 3.4505 | 0.0001 | NEK2 | 1q32.2-q41 |
| 203560_at | 3.2844 | 0.0000 | GGH | 8q12.3 |
| 204822_at | 3.2671 | 0.0000 | TTK | 6q13-q21 |
| 218009_s_at | 2.9870 | 0.0007 | PRC1 | 15q26.1 |
| 219787_s_at | 2.9277 | 0.0000 | ECT2 | 3q26.1-q26.2 |
| 207828_s_at | 2.8762 | 0.0000 | CENPF | 1q32-q41 |
| 201292_at | 2.7419 | 0.0004 | TOP2A | 17q21-q22 |
| 202095_s_at | 2.7181 | 0.0002 | BIRC5 | 17q25 |
| 203362_s_at | 2.6665 | 0.0003 | MAD2L1 | 4q27 |
| 219918_s_at | 2.5857 | 0.0008 | ASPM | 1q31 |
| 210559_s_at | 2.5430 | 0.0009 | CDC2 | 10q21.1 |
| 218355_at | 2.4355 | 0.0002 | KIF4A | Xq13.1 |
| 218542_at | 2.3846 | 0.0002 | C10orf3 | 10q23.33 |
| 201897_s_at | 2.3630 | 0.0002 | CKS1B | 1q21.2 |
| 204170_s_at | 2.3618 | 0.0006 | CKS2 | 9q22 |
| 222077_s_at | 2.3545 | 0.0003 | RACGAP1 | 12q13.12 |
| 202580_x_at | 2.3527 | 0.0002 | FOXM1 | 12p13 |
| 204962_s_at | 2.3105 | 0.0006 | CENPA | 2p24-p21 |
| 203214_x_at | 2.2898 | 0.0006 | CDC2 | 10q21.1 |
| 219306_at | 2.2171 | 0.0002 | KIF15 | 3p21.31 |
| 218755_at | 2.2094 | 0.0000 | KIF20A | 5q31 |
| 204203_at | 2.2087 | 0.0007 | CEBPG | 19q13.11 |
| 209642_at | 2.1682 | 0.0009 | BUB1 | 2q14 |
| 201761_at | 2.1523 | 0.0001 | MTHFD2 | 2p13.1 |
| 204127_at | 2.0969 | 0.0007 | RFC3 | 13q12.3-q13 |
| 210052_s_at | 2.0621 | 0.0006 | TPX2 | 20q11.2 |
| 209408_at | 2.0434 | 0.0007 | KIF2C | 1p34.1 |
| 201848_s_at | 2.0216 | 0.0003 | BNIP3 | 10q26.3 |
| 203418_at | 1.9936 | 0.0000 | CCNA2 | 4q25-q31 |
| 220651_s_at | 1.9810 | 0.0009 | MCM10 | 10p13 |
| 204092_s_at | 1.9733 | 0.0002 | STK6 | 20q13.2-q13.3 |
| 204146_at | 1.9688 | 0.0009 | RAD51AP1 | 12p13.2-p13.1 |
| 212020_s_at | 1.9509 | 0.0000 | MKI67 | 10q25-qter |
| 214007_s_at | 1.9251 | 0.0010 | PTK9 | 12q12 |
| 211762_s_at | 1.9227 | 0.0008 | KPNA2 | 17q23.1-q23.3 |
| 218662_s_at | 1.9168 | 0.0009 | HCAP-G | 4p15.33 |
| 219004_s_at | 1.8467 | 0.0001 | C21orf45 | 21q22.11 |
| 209172_s_at | 1.8223 | 0.0008 | CENPF | 1q32-q41 |
| 220295_x_at | 1.8194 | 0.0001 | DEPDC1 | 1p31.2 |
| 201088_at | 1.8150 | 0.0000 | KPNA2 | 17q23.1-q23.3 |
| 211519_s_at | 1.7827 | 0.0004 | KIF2C | 1p34.1 |
| 218349_s_at | 1.7708 | 0.0008 | ZWILCH | 15q22.31 |
| 201635_s_at | 1.7596 | 0.0002 | FXR1 | 3q28 |
| 201636_at | 1.7548 | 0.0006 | FXR1 | 3q28 |
| 218252_at | 1.7535 | 0.0008 | CKAP2 | 13q14 |
| 204887_s_at | 1.7438 | 0.0001 | PLK4 | 4q27-q28 |
| 222039_at | 1.7429 | 0.0004 | LOC146909 | 17q21.31 |
| 200841_s_at | 1.7162 | 0.0000 | EPRS | 1q41-q42 |
| 201637_s_at | 1.6647 | 0.0001 | FXR1 | 3q28 |
| 212023_s_at | 1.6156 | 0.0004 | MKI67 | 10q25-qter |
| 203017_s_at | 1.5866 | 0.0000 | SSX2IP | 1p22.3 |
| 212290_at | 1.5753 | 0.0000 | SLC7A1 | 13q12-q14 |
| 213189_at | 1.5745 | 0.0006 | DKFZp667G2110 | 3q11.2 |
| 211080_s_at | 1.5743 | 0.0001 | NEK2 | 1q32.2-q41 |
| 209709_s_at | 1.5735 | 0.0003 | HMMR | 5q33.2-qter |
| 204649_at | 1.5508 | 0.0007 | TROAP | 12q13.12 |
| 203016_s_at | 1.5441 | 0.0001 | SSX2IP | 1p22.3 |
| 206686_at | 1.5379 | 0.0000 | PDK1 | 2q31.1 |
| 209753_s_at | 1.5346 | 0.0002 | TMPO | 12q22 |
| 212789_at | 1.5284 | 0.0004 | hCAP-D3 | 11q25 |
| 209257_s_at | 1.5280 | 0.0010 | CSPG6 | 10q25 |
| 208777_s_at | 1.5128 | 0.0008 | PSMD11 | 17q11.2 |
| 201606_s_at | 1.5045 | 0.0009 | PWP1 | 12q23.3 |

**Supplementary Table 2B**

**Current/Never (CN) comparison, early stage Tumor (T) tissues: down-regulated probes**

| **Probe ID** | **CN**  **Fold Change < 0.6667** | **CN**  **p-value < 0.001** | **Gene Symbol** | **Chromosomal Location** |
| --- | --- | --- | --- | --- |
| 203757_s_at | 0.1980 | 0.0007 | CEACAM6 | 19q13.2 |
| 209373_at | 0.2471 | 0.0001 | MALL | 2q13 |
| 212950_at | 0.2683 | 0.0008 | GPR116 | 6p12.3 |
| 212951_at | 0.3126 | 0.0004 | GPR116 | 6p12.3 |
| 208891_at | 0.3249 | 0.0009 | DUSP6 | 12q22-q23 |
| 201286_at | 0.3355 | 0.0000 | SDC1 | 2p24.1 |
| 208893_s_at | 0.3409 | 0.0003 | DUSP6 | 12q22-q23 |
| 218211_s_at | 0.3448 | 0.0000 | MLPH | 2q37.3 |
| 203571_s_at | 0.3748 | 0.0006 | C10orf116 | 10q23.2 |
| 209292_at | 0.4052 | 0.0007 | ID4 | 6p22-p21 |
| 204802_at | 0.4179 | 0.0001 | RRAD | 16q22 |
| 201581_at | 0.4229 | 0.0007 | TXNDC13 | 20p12 |
| 212256_at | 0.4250 | 0.0003 | GALNT10 | 5q33.2 |
| 217967_s_at | 0.4260 | 0.0006 | C1orf24 | 1q25 |
| 205200_at | 0.4270 | 0.0003 | CLEC3B | 3p22-p21.3 |
| 210674_s_at | 0.4273 | 0.0001 | PCDHA12 | 5q31 |
| 201360_at | 0.4282 | 0.0002 | CST3 | 20p11.21 |
| 219206_x_at | 0.4350 | 0.0000 | TMBIM4 | 12q14.1-q15 |
| 200973_s_at | 0.4362 | 0.0004 | TSPAN3 | 15q24.3 |
| 203227_s_at | 0.4385 | 0.0000 | TSPAN31 | 12q13.3 |
| 205539_at | 0.4438 | 0.0003 | AVIL | 12q14.1 |
| 200766_at | 0.4506 | 0.0003 | CTSD | 11p15.5 |
| 204803_s_at | 0.4532 | 0.0001 | RRAD | 16q22 |
| 209605_at | 0.4550 | 0.0003 | TST | 22q13.1 |
| 215399_s_at | 0.4561 | 0.0002 | OS9 | 12q13 |
| 208890_s_at | 0.4623 | 0.0000 | PLXNB2 | 22q13.33 |
| 212473_s_at | 0.4665 | 0.0005 | MICAL2 | 11p15.3 |
| 217798_at | 0.4689 | 0.0005 | CNOT2 | 12q15 |
| 206170_at | 0.4699 | 0.0000 | ADRB2 | 5q31-q32 |
| 208703_s_at | 0.4718 | 0.0001 | APLP2 | 11q23-q25|11q24 |
| 201061_s_at | 0.4752 | 0.0000 | STOM | 9q34.1 |
| 202071_at | 0.4802 | 0.0005 | SDC4 | 20q12 |
| 201651_s_at | 0.4883 | 0.0002 | PACSIN2 | 22q13.2-13.33 |
| 204306_s_at | 0.4939 | 0.0003 | CD151 | 11p15.5 |
| 206528_at | 0.4984 | 0.0000 | TRPC6 | 11q21-q22 |
| 211404_s_at | 0.4989 | 0.0007 | APLP2 | 11q23-q25|11q24 |
| 208873_s_at | 0.5035 | 0.0001 | C5orf18 | 5q22-q23 |
| 219909_at | 0.5111 | 0.0003 | MMP28 | 17q11-q21.1 |
| 209264_s_at | 0.5138 | 0.0003 | TSPAN4 | 11p15.5 |
| 201287_s_at | 0.5145 | 0.0007 | SDC1 | 2p24.1 |
| 212472_at | 0.5152 | 0.0010 | MICAL2 | 11p15.3 |
| 213880_at | 0.5165 | 0.0006 | LGR5 | 12q22-q23 |
| 208704_x_at | 0.5168 | 0.0000 | APLP2 | 11q23-q25|11q24 |
| 200972_at | 0.5234 | 0.0003 | TSPAN3 | 15q24.3 |
| 201655_s_at | 0.5267 | 0.0004 | HSPG2 | 1p36.1-p34 |
| 203226_s_at | 0.5277 | 0.0000 | TSPAN31 | 12q13.3 |
| 210314_x_at | 0.5306 | 0.0009 | TNFSF13 | 17p13.1 |
| 208760_at | 0.5307 | 0.0001 | UBE2I | 16p13.3 |
| 221127_s_at | 0.5337 | 0.0006 | RIG | 11p15.1 |
| 202284_s_at | 0.5357 | 0.0003 | CDKN1A | 6p21.2 |
| 210788_s_at | 0.5378 | 0.0003 | DHRS7 | 14q23.1 |
| 204276_at | 0.5396 | 0.0002 | TK2 | 16q22-q23.1 |
| 212334_at | 0.5407 | 0.0005 | GNS | 12q14 |
| 206114_at | 0.5408 | 0.0002 | EPHA4 | 2q36.1 |
| 202739_s_at | 0.5433 | 0.0002 | PHKB | 16q12-q13 |
| 200714_x_at | 0.5459 | 0.0001 | OS9 | 12q13 |
| 208702_x_at | 0.5474 | 0.0006 | APLP2 | 11q23-q25|11q24 |
| 205559_s_at | 0.5513 | 0.0008 | PCSK5 | 9q21.3 |
| 208248_x_at | 0.5519 | 0.0000 | APLP2 | 11q23-q25|11q24 |
| 218368_s_at | 0.5526 | 0.0003 | TNFRSF12A | 16p13.3 |
| 200696_s_at | 0.5528 | 0.0003 | GSN | 9q33 |
| 201331_s_at | 0.5548 | 0.0007 | STAT6 | 12q13 |
| 212576_at | 0.5590 | 0.0002 | MGRN1 | 16p13.3 |
| 202068_s_at | 0.5605 | 0.0009 | LDLR | 19p13.3 |
| 211998_at | 0.5644 | 0.0005 | H3F3B | 17q25 |
| 201809_s_at | 0.5646 | 0.0003 | ENG | 9q33-q34.1 |
| 204916_at | 0.5668 | 0.0006 | RAMP1 | 2q36-q37.1 |
| 205931_s_at | 0.5669 | 0.0003 | CREB5 | 7p15.1 |
| 220622_at | 0.5683 | 0.0002 | LRRC31 | 3q26.2 |
| 212589_at | 0.5703 | 0.0005 | RRAS2 | 11p15.2 |
| 204862_s_at | 0.5740 | 0.0006 | NME3 | 16q13 |
| 209499_x_at | 0.5791 | 0.0004 | TNFSF13 | 17p13.1 |
| 209263_x_at | 0.5792 | 0.0003 | TSPAN4 | 11p15.5 |
| 201341_at | 0.5819 | 0.0009 | ENC1 | 5q12-q13.3 |
| 209513_s_at | 0.5857 | 0.0001 | HSDL2 | 9q32 |
| 209667_at | 0.5969 | 0.0000 | CES2 | 16q22.1 |
| 200810_s_at | 0.5974 | 0.0005 | CIRBP | 19p13.3 |
| 212622_at | 0.6018 | 0.0001 | TMEM41B | 11p15.4 |
| 208634_s_at | 0.6019 | 0.0001 | MACF1 | 1p32-p31 |
| 212914_at | 0.6033 | 0.0007 | CBX7 | 22q13.1 |
| 215684_s_at | 0.6080 | 0.0007 | ASCC2 | 22q12.1 |
| 212071_s_at | 0.6114 | 0.0005 | SPTBN1 | 2p21 |
| 214841_at | 0.6132 | 0.0006 | CNIH3 | 1q42.12 |
| 221489_s_at | 0.6143 | 0.0004 | SPRY4 | 5q31.3 |
| 218686_s_at | 0.6321 | 0.0001 | RHBDF1 | 16p13.3 |
| 217287_s_at | 0.6366 | 0.0009 | TRPC6 | 11q21-q22 |
| 210844_x_at | 0.6372 | 0.0001 | CTNNA1 | 5q31 |
| 200675_at | 0.6406 | 0.0001 | CD81 | 11p15.5 |
| 213244_at | 0.6469 | 0.0001 | SCAMP4 | 19p13.3 |
| 205717_x_at | 0.6492 | 0.0004 | PCDHGC3 | 5q31 |
| 200621_at | 0.6514 | 0.0007 | CSRP1 | 1q32 |
| 218679_s_at | 0.6522 | 0.0005 | VPS28 | 8q24.3 |
| 214894_x_at | 0.6524 | 0.0003 | MACF1 | 1p32-p31 |
| 221756_at | 0.6553 | 0.0003 | MGC17330 | 22q12.2 |
| 200678_x_at | 0.6571 | 0.0010 | GRN | 17q21.32 |
| 210507_s_at | 0.6576 | 0.0008 | AVIL | 12q14.1 |
| 201282_at | 0.6602 | 0.0010 | OGDH | 7p14-p13 |
| 221519_at | 0.6647 | 0.0006 | FBXW4 | 10q24 |

**Supplementary Table 2C.**

**Current/Never (CN) comparison, late stage Tumor tissues: up + down-regulated probes**

| **Probe ID** | **CN  Fold Change**  **>1.5**  **or <0.6667** | **CN**  **p-value** | **Gene Symbol** | **Chromosomal Location** |
| --- | --- | --- | --- | --- |
| 201437_s_at | 2.0413 | 0.0000 | EIF4E | 4q21-q25 |
| 212698_s_at | 2.6607 | 0.0001 | 39335 | 2q13 |
| 209498_at | 0.4759 | 0.0002 | CEACAM1 | 19q13.2 |
| 204973_at | 0.6063 | 0.0002 | GJB1 | Xq13.1 |
| 204004_at | 2.2310 | 0.0002 | PAWR | 12q21 |
| 203343_at | 3.0663 | 0.0002 | UGDH | 4p15.1 |
| 206055_s_at | 1.8010 | 0.0003 | SNRPA1 | 15q26.3 |
| 212795_at | 1.5503 | 0.0005 | KIAA1033 | 12q24.11 |
| 214059_at | 0.5261 | 0.0005 | IFI44 | 1p31.1 |
| 208762_at | 1.5088 | 0.0005 | SUMO1 | 2q33 |
| 217887_s_at | 1.5433 | 0.0005 | EPS15 | 1p32 |
| 204340_at | 0.6635 | 0.0006 | CXorf12 | Xq28 |
| 222016_s_at | 0.5787 | 0.0006 | ZNF323 |  |
| 201624_at | 2.0285 | 0.0007 | DARS | 2q21.3 |
| 219237_s_at | 1.7811 | 0.0007 | DNAJB14 | 4q23 |
| 205229_s_at | 2.9992 | 0.0007 | COCH | 14q12-q13 |
| 218351_at | 1.5367 | 0.0007 | COMMD8 | 4p12 |
| 203302_at | 2.0269 | 0.0008 | DCK | 4q13.3-q21.1 |
| 218795_at | 0.6087 | 0.0008 | ACP6 | 1q21 |
| 209135_at | 2.7417 | 0.0010 | ASPH | 8q12.1 |

**Supplementary Table 2D.**

**Gene Ontology (GO) functional categories for the Current/Never (C/N) smoker comparison (up and down-regulated genes) in early stage Tumor (T) tissue (p<0.01, Fisher’s exact test)**

| **GO ID** | **Total # genes classified in U133A** | **Down regulated** | **Up regulated** | **Change** | **p-value Down** | **p-value Up** | **p-value Overall changes** | **GO categories** |
| --- | --- | --- | --- | --- | --- | --- | --- | --- |
| 44430 | 332 | 1 | 13 | 14 | 0.9051 | 0.0000 | 0.0000 | cytoskeletal part |
| 51301 | 129 | 0 | 12 | 12 | 1.0000 | 0.0000 | 0.0000 | cell division |
| 7094 | 5 | 0 | 3 | 3 | 1.0000 | 0.0000 | 0.0000 | mitotic spindle checkpoint |
| 7093 | 14 | 0 | 5 | 5 | 1.0000 | 0.0000 | 0.0000 | mitotic checkpoint |
| 7088 | 40 | 0 | 6 | 6 | 1.0000 | 0.0000 | 0.0000 | regulation of mitosis |
| 7067 | 127 | 0 | 12 | 12 | 1.0000 | 0.0000 | 0.0000 | mitosis |
| 7049 | 601 | 3 | 20 | 23 | 0.7968 | 0.0000 | 0.0000 | cell cycle |
| 7017 | 104 | 0 | 8 | 8 | 1.0000 | 0.0000 | 0.0000 | microtubule-based process |
| 7010 | 291 | 4 | 9 | 13 | 0.1440 | 0.0000 | 0.0000 | cytoskeleton organization and biogenesis |
| 5856 | 601 | 8 | 13 | 21 | 0.0563 | 0.0000 | 0.0000 | cytoskeleton |
| 5819 | 35 | 0 | 7 | 7 | 1.0000 | 0.0000 | 0.0000 | spindle |
| 775 | 26 | 0 | 6 | 6 | 1.0000 | 0.0000 | 0.0000 | chromosome, pericentric region |
| 31577 | 5 | 0 | 3 | 3 | 1.0000 | 0.0000 | 0.0000 | spindle checkpoint |
| 279 | 161 | 0 | 13 | 13 | 1.0000 | 0.0000 | 0.0000 | M phase |
| 278 | 177 | 0 | 14 | 14 | 1.0000 | 0.0000 | 0.0000 | mitotic cell cycle |
| 43232 | 1014 | 9 | 20 | 29 | 0.2707 | 0.0000 | 0.0000 | intracellular non-membrane-bound organelle |
| 43228 | 1014 | 9 | 20 | 29 | 0.2707 | 0.0000 | 0.0000 | non-membrane-bound organelle |
| 87 | 129 | 0 | 12 | 12 | 1.0000 | 0.0000 | 0.0000 | M phase of mitotic cell cycle |
| 15630 | 188 | 0 | 13 | 13 | 1.0000 | 0.0000 | 0.0000 | microtubule cytoskeleton |
| 6996 | 666 | 6 | 13 | 19 | 0.3174 | 0.0000 | 0.0001 | organelle organization and biogenesis |
| 16538 | 2 | 0 | 2 | 2 | 1.0000 | 0.0000 | 0.0001 | cyclin-dependent protein kinase regulator activity |
| 5874 | 131 | 0 | 8 | 8 | 1.0000 | 0.0000 | 0.0001 | microtubule |
| 5694 | 212 | 1 | 9 | 10 | 0.7758 | 0.0000 | 0.0001 | chromosome |
| 75 | 43 | 0 | 5 | 5 | 1.0000 | 0.0000 | 0.0001 | cell cycle checkpoint |
| 8283 | 488 | 6 | 9 | 15 | 0.1231 | 0.0002 | 0.0003 | cell proliferation |
| 5876 | 13 | 0 | 3 | 3 | 1.0000 | 0.0000 | 0.0004 | spindle microtubule |
| 51726 | 415 | 3 | 10 | 13 | 0.5559 | 0.0000 | 0.0007 | regulation of cell cycle |
| 44427 | 173 | 1 | 7 | 8 | 0.7041 | 0.0000 | 0.0007 | chromosomal part |
| 51319 | 4 | 0 | 2 | 2 | 1.0000 | 0.0001 | 0.0007 | G2 phase |
| 776 | 16 | 0 | 3 | 3 | 1.0000 | 0.0000 | 0.0007 | kinetochore |
| 85 | 4 | 0 | 2 | 2 | 1.0000 | 0.0001 | 0.0007 | G2 phase of mitotic cell cycle |
| 74 | 414 | 3 | 10 | 13 | 0.5542 | 0.0000 | 0.0007 | regulation of progression through cell cycle |
| 3777 | 39 | 0 | 4 | 4 | 1.0000 | 0.0000 | 0.0009 | microtubule motor activity |
| 7051 | 19 | 0 | 3 | 3 | 1.0000 | 0.0001 | 0.0012 | spindle organization and biogenesis |
| 50871 | 5 | 2 | 0 | 2 | 0.0005 | 1.0000 | 0.0012 | positive regulation of B cell activation |
| 8092 | 285 | 8 | 2 | 10 | 0.0008 | 0.3427 | 0.0013 | cytoskeletal protein binding |
| 19887 | 48 | 2 | 2 | 4 | 0.0438 | 0.0176 | 0.0020 | protein kinase regulator activity |
| 51693 | 23 | 3 | 0 | 3 | 0.0005 | 1.0000 | 0.0021 | actin filament capping |
| 51016 | 23 | 3 | 0 | 3 | 0.0005 | 1.0000 | 0.0021 | barbed-end actin filament capping |
| 30835 | 24 | 3 | 0 | 3 | 0.0006 | 1.0000 | 0.0023 | negative regulation of actin filament depolymerization |
| 30834 | 24 | 3 | 0 | 3 | 0.0006 | 1.0000 | 0.0023 | regulation of actin filament depolymerization |
| 30042 | 24 | 3 | 0 | 3 | 0.0006 | 1.0000 | 0.0023 | actin filament depolymerization |
| 51329 | 54 | 0 | 4 | 4 | 1.0000 | 0.0001 | 0.0031 | interphase of mitotic cell cycle |
| 51325 | 55 | 0 | 4 | 4 | 1.0000 | 0.0001 | 0.0033 | interphase |
| 50864 | 8 | 2 | 0 | 2 | 0.0013 | 1.0000 | 0.0033 | regulation of B cell activation |
| 42113 | 28 | 2 | 1 | 3 | 0.0160 | 0.1126 | 0.0037 | B cell activation |
| 7018 | 57 | 0 | 4 | 4 | 1.0000 | 0.0001 | 0.0037 | microtubule-based movement |
| 19207 | 60 | 2 | 2 | 4 | 0.0652 | 0.0267 | 0.0045 | kinase regulator activity |
| 48522 | 511 | 8 | 5 | 13 | 0.0250 | 0.0642 | 0.0046 | positive regulation of cellular process |
| 51261 | 32 | 3 | 0 | 3 | 0.0014 | 1.0000 | 0.0054 | protein depolymerization |
| 45859 | 144 | 4 | 2 | 6 | 0.0178 | 0.1247 | 0.0055 | regulation of protein kinase activity |
| 43549 | 145 | 4 | 2 | 6 | 0.0182 | 0.1261 | 0.0057 | regulation of kinase activity |
| 51338 | 146 | 4 | 2 | 6 | 0.0186 | 0.1275 | 0.0059 | regulation of transferase activity |
| 51129 | 33 | 3 | 0 | 3 | 0.0015 | 1.0000 | 0.0059 | negative regulation of cell organization and biogenesis |
| 5875 | 69 | 0 | 4 | 4 | 1.0000 | 0.0002 | 0.0074 | microtubule associated complex |
| 7052 | 12 | 0 | 2 | 2 | 1.0000 | 0.0011 | 0.0076 | mitotic spindle organization and biogenesis |
| 910 | 12 | 0 | 2 | 2 | 1.0000 | 0.0011 | 0.0076 | cytokinesis |
| 30705 | 70 | 0 | 4 | 4 | 1.0000 | 0.0002 | 0.0078 | cytoskeleton-dependent intracellular transport |
| 79 | 37 | 1 | 2 | 3 | 0.2280 | 0.0107 | 0.0081 | regulation of cyclin-dependent protein kinase activity |
| 8064 | 37 | 3 | 0 | 3 | 0.0021 | 1.0000 | 0.0081 | regulation of actin polymerization and/or depolymerization |
| 51098 | 13 | 1 | 1 | 2 | 0.0868 | 0.0539 | 0.0090 | regulation of binding |
| 5515 | 3847 | 31 | 25 | 56 | 0.1795 | 0.0062 | 0.0090 | protein binding |
| 30832 | 40 | 3 | 0 | 3 | 0.0027 | 1.0000 | 0.0100 | regulation of actin filament length |
| 51242 | 439 | 7 | 4 | 11 | 0.0324 | 0.1150 | 0.0101 | positive regulation of cellular physiological process |
| 922 | 14 | 0 | 2 | 2 | 1.0000 | 0.0016 | 0.0104 | spindle pole |
| 44446 | 1468 | 5 | 21 | 26 | 0.9818 | 0.0000 | 0.0107 | intracellular organelle part |
| 44422 | 1468 | 5 | 21 | 26 | 0.9818 | 0.0000 | 0.0107 | organelle part |
| 6433 | 1 | 0 | 1 | 1 | 1.0000 | 0.0043 | 0.0112 | prolyl-tRNA aminoacylation |
| 4941 | 1 | 1 | 0 | 1 | 0.0070 | 1.0000 | 0.0112 | beta2-adrenergic receptor activity |
| 4827 | 1 | 0 | 1 | 1 | 1.0000 | 0.0043 | 0.0112 | proline-tRNA ligase activity |
| 9353 | 1 | 1 | 0 | 1 | 0.0070 | 1.0000 | 0.0112 | oxoglutarate dehydrogenase complex (sensu Eukaryota) |
| 4487 | 1 | 0 | 1 | 1 | 1.0000 | 0.0043 | 0.0112 | methylenetetrahydrofolate dehydrogenase (NAD+) activity |
| 4192 | 1 | 1 | 0 | 1 | 0.0070 | 1.0000 | 0.0112 | cathepsin D activity |
| 45837 | 1 | 0 | 1 | 1 | 1.0000 | 0.0043 | 0.0112 | negative regulation of membrane potential |
| 7108 | 1 | 0 | 1 | 1 | 1.0000 | 0.0043 | 0.0112 | cytokinesis, initiation of separation |
| 940 | 1 | 0 | 1 | 1 | 1.0000 | 0.0043 | 0.0112 | outer kinetochore of condensed chromosome |
| 920 | 1 | 0 | 1 | 1 | 1.0000 | 0.0043 | 0.0112 | cell separation during cytokinesis |
| 915 | 1 | 0 | 1 | 1 | 1.0000 | 0.0043 | 0.0112 | cytokinesis, contractile ring formation |
| 912 | 1 | 0 | 1 | 1 | 1.0000 | 0.0043 | 0.0112 | cytokinesis, formation of actomyosin apparatus |
| 43353 | 1 | 0 | 1 | 1 | 1.0000 | 0.0043 | 0.0112 | enucleate erythrocyte differentiation |
| 72 | 1 | 0 | 1 | 1 | 1.0000 | 0.0043 | 0.0112 | M phase specific microtubule process |
| 43128 | 1 | 1 | 0 | 1 | 0.0070 | 1.0000 | 0.0112 | positive regulation of 1-phosphatidylinositol 4-kinase activity |
| 43126 | 1 | 1 | 0 | 1 | 0.0070 | 1.0000 | 0.0112 | regulation of 1-phosphatidylinositol 4-kinase activity |
| 46813 | 1 | 1 | 0 | 1 | 0.0070 | 1.0000 | 0.0112 | virion attachment, binding of host cell surface receptor |
| 8464 | 1 | 0 | 1 | 1 | 1.0000 | 0.0043 | 0.0112 | gamma-glutamyl hydrolase activity |
| 8449 | 1 | 1 | 0 | 1 | 0.0070 | 1.0000 | 0.0112 | N-acetylglucosamine-6-sulfatase activity |
| 15281 | 1 | 1 | 0 | 1 | 0.0070 | 1.0000 | 0.0112 | nonselective cation channel activity |
| 15249 | 1 | 1 | 0 | 1 | 0.0070 | 1.0000 | 0.0112 | nonselective channel activity |
| 42803 | 79 | 2 | 2 | 4 | 0.1045 | 0.0443 | 0.0118 | protein homodimerization activity |
| 5871 | 15 | 0 | 2 | 2 | 1.0000 | 0.0018 | 0.0119 | kinesin complex |
| 3682 | 43 | 1 | 2 | 3 | 0.2597 | 0.0143 | 0.0122 | chromatin binding |
| 15631 | 43 | 1 | 2 | 3 | 0.2597 | 0.0143 | 0.0122 | tubulin binding |
| 43119 | 457 | 7 | 4 | 11 | 0.0390 | 0.1280 | 0.0134 | positive regulation of physiological process |
| 226 | 45 | 0 | 3 | 3 | 1.0000 | 0.0009 | 0.0138 | microtubule cytoskeleton organization and biogenesis |
| 8284 | 127 | 4 | 1 | 5 | 0.0117 | 0.4198 | 0.0140 | positive regulation of cell proliferation |
| 16043 | 1211 | 9 | 13 | 22 | 0.4701 | 0.0011 | 0.0146 | cell organization and biogenesis |
| 8154 | 46 | 3 | 0 | 3 | 0.0040 | 1.0000 | 0.0147 | actin polymerization and/or depolymerization |
| 30036 | 129 | 4 | 1 | 5 | 0.0123 | 0.4248 | 0.0149 | actin cytoskeleton organization and biogenesis |

**Supplementary table 2E.**

**Current/Never (C/N) and Former/Never (F/N) comparisons in early stage Tumor (T) tissue: overlapping probe list with p<0.001 and fold change >1.5 for both C/N and F/N comparisons**

| **Probe ID** | **Gene** | **Chromosomal** |  | **Current / Never** | |  | **Former / Never** | |
| --- | --- | --- | --- | --- | --- | --- | --- | --- |
|  | **Symbol** | **Location** |  | **Fold Change** | **P-value** |  | **Fold Change** | **P-value** |
| 206170_at | ADRB2 | 5q31-q32 |  | 0.4699 | 0.0000 |  | 0.5257 | 0.0002 |
| 208248_x_at | APLP2 | 11q23-q25|11q24 |  | 0.5519 | 0.0000 |  | 0.5312 | 0.0001 |
| 208703_s_at | APLP2 | 11q23-q25|11q24 |  | 0.4718 | 0.0001 |  | 0.4517 | 0.0005 |
| 208704_x_at | APLP2 | 11q23-q25|11q24 |  | 0.5168 | 0.0000 |  | 0.5193 | 0.0001 |
| 205539_at | AVIL | 12q14.1 |  | 0.4438 | 0.0003 |  | 0.3810 | 0.0004 |
| 209667_at | CES2 | 16q22.1 |  | 0.5969 | 0.0000 |  | 0.6058 | 0.0002 |
| 210788_s_at | DHRS7 | 14q23.1 |  | 0.5378 | 0.0003 |  | 0.5060 | 0.0009 |
| 212334_at | GNS | 12q14 |  | 0.5407 | 0.0005 |  | 0.4505 | 0.0002 |
| 209513_s_at | HSDL2 | 9q32 |  | 0.5857 | 0.0001 |  | 0.5706 | 0.0005 |
| 218755_at | **KIF20A** | 5q31 |  | 2.2094 | 0.0000 |  | 2.0606 | 0.0010 |
| 208634_s_at | MACF1 | 1p32-p31 |  | 0.6019 | 0.0001 |  | 0.5825 | 0.0004 |
| 212023_s_at | **MKI67** | 10q25-qter |  | 1.6156 | 0.0004 |  | 1.7318 | 0.0006 |
| 200714_x_at | OS9 | 12q13 |  | 0.5459 | 0.0001 |  | 0.5059 | 0.0003 |
| 215399_s_at | OS9 | 12q13 |  | 0.4561 | 0.0002 |  | 0.4333 | 0.0009 |
| 201651_s_at | PACSIN2 | 22q13.2-13.33 |  | 0.4883 | 0.0002 |  | 0.4536 | 0.0005 |
| 205559_s_at | PCSK5 | 9q21.3 |  | 0.5513 | 0.0008 |  | 0.4461 | 0.0002 |
| 206686_at | **PDK1** | 2q31.1 |  | 1.5379 | 0.0000 |  | 1.5828 | 0.0002 |
| 221127_s_at | RIG | 11p15.1 |  | 0.5337 | 0.0006 |  | 0.4827 | 0.0009 |
| 204802_at | RRAD | 16q22 |  | 0.4179 | 0.0001 |  | 0.3330 | 0.0001 |
| 204803_s_at | RRAD | 16q22 |  | 0.4532 | 0.0001 |  | 0.4311 | 0.0005 |
| 203017_s_at | **SSX2IP** | 1p22.3 |  | 1.5866 | 0.0000 |  | 1.6630 | 0.0000 |
| 201061_s_at | STOM | 9q34.1 |  | 0.4752 | 0.0000 |  | 0.4840 | 0.0001 |
| 212622_at | TMEM41B | 11p15.4 |  | 0.6018 | 0.0001 |  | 0.5766 | 0.0003 |
| 206528_at | TRPC6 | 11q21-q22 |  | 0.4984 | 0.0000 |  | 0.4927 | 0.0002 |
| 203226_s_at | TSPAN31 | 12q13.3 |  | 0.5277 | 0.0000 |  | 0.5606 | 0.0009 |
| 203227_s_at | TSPAN31 | 12q13.3 |  | 0.4385 | 0.0000 |  | 0.4302 | 0.0003 |

In **bold**, up-regulated genes

**Supplementary table 2F.**

**Gene list from GSEA-comparison of up-regulated C/N genes and F/N genes in early stage Tumor (T) tissues**

| **Probe ID** | **Gene Symbol** | **Core enrichment** | **GSEA index** |
| --- | --- | --- | --- |
| 203017_s_at | SSX2IP | YES | 1 |
| 218542_at | C10orf3 | YES | 2 |
| 201292_at | TOP2A | YES | 3 |
| 209709_s_at | HMMR | YES | 4 |
| 204887_s_at | PLK4 | YES | 5 |
| 207828_s_at | CENPF | YES | 6 |
| 219787_s_at | ECT2 | YES | 7 |
| 201088_at | KPNA2 | YES | 8 |
| 204092_s_at | STK6 | YES | 9 |
| 222039_at | LOC146909 | YES | 10 |
| 209753_s_at | TMPO | YES | 11 |
| 204641_at | NEK2 | YES | 12 |
| 219918_s_at | ASPM | YES | 13 |
| 210052_s_at | TPX2 | YES | 14 |
| 203418_at | CCNA2 | YES | 15 |
| 201291_s_at | TOP2A | YES | 16 |
| 218355_at | KIF4A | YES | 17 |
| 218009_s_at | PRC1 | YES | 18 |
| 211519_s_at | KIF2C | YES | 19 |
| 222077_s_at | RACGAP1 | YES | 20 |
| 212020_s_at | MKI67 | YES | 21 |
| 209408_at | KIF2C | YES | 22 |
| 203214_x_at | CDC2 | YES | 23 |
| 201897_s_at | CKS1B | YES | 24 |
| 212023_s_at | MKI67 | YES | 25 |
| 203362_s_at | MAD2L1 | YES | 26 |
| 211762_s_at | KPNA2 | YES | 27 |
| 204822_at | TTK | YES | 28 |
| 220295_x_at | DEPDC1 | YES | 29 |
| 201761_at | MTHFD2 | YES | 30 |
| 219306_at | KIF15 | YES | 31 |
| 214007_s_at | PTK9 | YES | 32 |
| 202095_s_at | BIRC5 | YES | 33 |
| 209642_at | BUB1 | YES | 34 |
| 211080_s_at | NEK2 | YES | 35 |
| 218755_at | KIF20A | YES | 36 |
| 204170_s_at | CKS2 | YES | 37 |
| 204962_s_at | CENPA | YES | 38 |
| 202580_x_at | FOXM1 | YES | 39 |
| 210559_s_at | CDC2 | YES | 40 |
| 200841_s_at | EPRS | YES | 41 |
| 204146_at | RAD51AP1 | YES | 42 |
| 212290_at | SLC7A1 | YES | 43 |
| 203560_at | GGH | YES | 44 |
| 203016_s_at | SSX2IP | YES | 45 |
| 201848_s_at | BNIP3 | YES | 46 |
| 218349_s_at | ZWILCH | YES | 47 |
| 201636_at | FXR1 | YES | 48 |
| 218252_at | CKAP2 | YES | 49 |
| 209172_s_at | CENPF | YES | 50 |
| 204649_at | TROAP | YES | 51 |
| 206686_at | PDK1 | YES | 52 |
| 212789_at | hCAP-D3 | YES | 53 |
| 208777_s_at | PSMD11 | YES | 54 |
| 220651_s_at | MCM10 | YES | 55 |
| 219004_s_at | C21orf45 | YES | 56 |
| 218662_s_at | HCAP-G | YES | 57 |
| 209257_s_at | CSPG6 | YES | 58 |
| 213189_at | DKFZp667G2110 | NO | 59 |
| 201606_s_at | PWP1 | NO | 60 |
| 204203_at | CEBPG | NO | 61 |
| 204127_at | RFC3 | NO | 62 |
| 201635_s_at | FXR1 | NO | 63 |
| 201637_s_at | FXR1 | NO | 64 |

**Supplementary table 2G.**

**Gene list from GSEA-comparison of down-regulated C/N genes and F/N genes in early stage Tumor (T) tissues**

| **Probe ID** | **Gene Symbol** | **Core enrichment** | **GSEA index** |
| --- | --- | --- | --- |
| 209667_at | CES2 | YES | 1 |
| 206170_at | ADRB2 | YES | 2 |
| 208760_at | UBE2I | YES | 3 |
| 208704_x_at | APLP2 | YES | 4 |
| 200714_x_at | OS9 | YES | 5 |
| 219909_at | MMP28 | YES | 6 |
| 203227_s_at | TSPAN31 | YES | 7 |
| 212622_at | TMEM41B | YES | 8 |
| 205559_s_at | PCSK5 | YES | 9 |
| 200810_s_at | CIRBP | YES | 10 |
| 220622_at | LRRC31 | YES | 11 |
| 215399_s_at | OS9 | YES | 12 |
| 203226_s_at | TSPAN31 | YES | 13 |
| 208248_x_at | APLP2 | YES | 14 |
| 211998_at | H3F3B | YES | 15 |
| 201286_at | SDC1 | YES | 16 |
| 209513_s_at | HSDL2 | YES | 17 |
| 215684_s_at | ASCC2 | YES | 18 |
| 206114_at | EPHA4 | YES | 19 |
| 208873_s_at | C5orf18 | YES | 20 |
| 202739_s_at | PHKB | YES | 21 |
| 212914_at | CBX7 | YES | 22 |
| 209373_at | MALL | YES | 23 |
| 204803_s_at | RRAD | YES | 24 |
| 219206_x_at | TMBIM4 | YES | 25 |
| 208703_s_at | APLP2 | YES | 26 |
| 212589_at | RRAS2 | YES | 27 |
| 205539_at | AVIL | YES | 28 |
| 204802_at | RRAD | YES | 29 |
| 205200_at | CLEC3B | YES | 30 |
| 201061_s_at | STOM | YES | 31 |
| 214841_at | CNIH3 | YES | 32 |
| 221489_s_at | SPRY4 | YES | 33 |
| 204276_at | TK2 | YES | 34 |
| 200675_at | CD81 | YES | 35 |
| 208634_s_at | MACF1 | YES | 36 |
| 211404_s_at | APLP2 | YES | 37 |
| 210788_s_at | DHRS7 | YES | 38 |
| 217967_s_at | C1orf24 | YES | 39 |
| 210507_s_at | AVIL | YES | 40 |
| 206528_at | TRPC6 | YES | 41 |
| 201809_s_at | ENG | YES | 42 |
| 204862_s_at | NME3 | YES | 43 |
| 217798_at | CNOT2 | YES | 44 |
| 221127_s_at | RIG | YES | 45 |
| 201651_s_at | PACSIN2 | YES | 46 |
| 212950_at | GPR116 | YES | 47 |
| 209605_at | TST | YES | 48 |
| 209292_at | ID4 | YES | 49 |
| 212256_at | GALNT10 | YES | 50 |
| 210674_s_at | PCDHA12 | YES | 51 |
| 212951_at | GPR116 | YES | 52 |
| 200696_s_at | GSN | YES | 53 |
| 203571_s_at | C10orf116 | YES | 54 |
| 208702_x_at | APLP2 | YES | 55 |
| 200766_at | CTSD | YES | 56 |
| 212334_at | GNS | YES | 57 |
| 200621_at | CSRP1 | YES | 58 |
| 218211_s_at | MLPH | YES | 59 |
| 204306_s_at | CD151 | YES | 60 |
| 221756_at | MGC17330 | YES | 61 |
| 202071_at | SDC4 | YES | 62 |
| 209263_x_at | TSPAN4 | YES | 63 |
| 201287_s_at | SDC1 | YES | 64 |
| 201655_s_at | HSPG2 | YES | 65 |
| 217287_s_at | TRPC6 | YES | 66 |
| 200678_x_at | GRN | YES | 67 |
| 221519_at | FBXW4 | YES | 68 |
| 208890_s_at | PLXNB2 | YES | 69 |
| 212473_s_at | MICAL2 | YES | 70 |
| 218368_s_at | TNFRSF12A | YES | 71 |
| 213880_at | LGR5 | YES | 72 |
| 200972_at | TSPAN3 | YES | 73 |
| 209264_s_at | TSPAN4 | YES | 74 |
| 208893_s_at | DUSP6 | YES | 75 |
| 201360_at | CST3 | YES | 76 |
| 213244_at | SCAMP4 | YES | 77 |
| 212576_at | MGRN1 | YES | 78 |
| 208891_at | DUSP6 | YES | 79 |
| 214894_x_at | MACF1 | YES | 80 |
| 212472_at | MICAL2 | YES | 81 |
| 200973_s_at | TSPAN3 | YES | 82 |
| 201581_at | TXNDC13 | YES | 83 |
| 210314_x_at | TNFSF13 | YES | 84 |
| 201341_at | ENC1 | YES | 85 |
| 212071_s_at | SPTBN1 | YES | 86 |
| 209499_x_at | TNFSF13 | YES | 87 |
| 202068_s_at | LDLR | YES | 88 |
| 218686_s_at | RHBDF1 | YES | 89 |
| 201331_s_at | STAT6 | YES | 90 |
| 205717_x_at | PCDHGC3 | NO | 91 |
| 218679_s_at | VPS28 | NO | 92 |
| 210844_x_at | CTNNA1 | NO | 93 |
| 205931_s_at | CREB5 | NO | 94 |
| 204916_at | RAMP1 | NO | 95 |
| 202284_s_at | CDKN1A | NO | 96 |
| 203757_s_at | CEACAM6 | NO | 97 |
| 201282_at | OGDH | NO | 98 |
