# Supplementary material for: Gene Expression Signature of Cigarette Smoking and Its Role in Lung Adenocarcinoma Development and Survival
Source: PLoS One. 2008 Feb 20;3(2):e1651. doi: 10.1371/journal.pone.0001651 (PMC2249927; doi:10.1371/journal.pone.0001651)
Supplement: Appendix S3 — Current/Never (C/N) smoking comparisons in Non-Tumor (NT) lung tissue. 3A Current/Never (C/N) comparison in Non-Tumor (NT) lung tissues: up-regulated probes. 3B Current/Never (C/N) comparison in Non-Tumor (NT) lung tissues: down-regulated probes . 3C Gene Ontology (GO) functional categories for the Current/Never (C/N) comparison (up and down-regulated genes) in Non-Tumor (NT) lung tissues. (0.21 MB DOC) [file pone.0001651.s003.doc]

**Appendix S3**

**Current/Never (C/N) smoking comparisons in Non-Tumor (NT) lung tissues**

**Supplementary Table 3A**

**Current/Never (C/N) comparison in Non-Tumor (NT) lung tissues: up-regulated probes**

| **Probe ID** | **Fold change > 1.5** | **CN p-value <0.001** | **Gene Symbol** | **Chromosomal Location** |
| --- | --- | --- | --- | --- |
| 219612_s_at | 5.4176 | 0.0000 | FGG | 4q28 |
| 202437_s_at | 5.2664 | 0.0000 | CYP1B1 | 2p21 |
| 202436_s_at | 3.9234 | 0.0000 | CYP1B1 | 2p21 |
| 202435_s_at | 2.9394 | 0.0000 | CYP1B1 | 2p21 |
| 205576_at | 2.8272 | 0.0000 | SERPIND1 | 22q11.2|22q11.21 |
| 220625_s_at | 2.2115 | 0.0001 | ELF5 | 11p13-p12 |
| 206420_at | 1.9467 | 0.0004 | IGSF6 | 16p12-p13 |
| 201884_at | 1.9090 | 0.0003 | CEACAM5 | 19q13.1-q13.2 |
| 205749_at | 1.8810 | 0.0006 | CYP1A1 | 15q22-q24 |
| 219890_at | 1.8630 | 0.0000 | CLEC5A | 7q33 |
| 204580_at | 1.8580 | 0.0000 | MMP12 | 11q22.3 |
| 203922_s_at | 1.8440 | 0.0002 | CYBB | Xp21.1 |
| 219837_s_at | 1.7605 | 0.0001 | CYTL1 | 4p16-p15 |
| 221266_s_at | 1.7330 | 0.0000 | TM7SF4 | 8q23 |
| 220380_at | 1.6903 | 0.0002 | DNASE2B | 1p22.3 |
| 211138_s_at | 1.6881 | 0.0008 | KMO | 1q42-q44 |
| 205306_x_at | 1.6711 | 0.0007 | KMO | 1q42-q44 |
| 205713_s_at | 1.6363 | 0.0004 | COMP | 19p13.1 |
| 220428_at | 1.6282 | 0.0000 | CD207 | 2p13 |
| 219725_at | 1.6249 | 0.0001 | TREM2 | 6p21.1 |
| 213695_at | 1.6132 | 0.0001 | PON3 | 7q21.3 |
| 221524_s_at | 1.5771 | 0.0001 | RRAGD | 6q15-q16 |
| 210325_at | 1.5697 | 0.0001 | CD1A | 1q22-q23 |
| 206914_at | 1.5661 | 0.0000 | CRTAM | 11q22-q23 |
| 205185_at | 1.5453 | 0.0000 | SPINK5 | 5q32 |
| 205738_s_at | 1.5441 | 0.0006 | FABP3 | 1p33-p32 |
| 210133_at | 1.5263 | 0.0002 | CCL11 | 17q21.1-q21.2 |
| 210184_at | 1.5177 | 0.0000 | ITGAX | 16p11.2 |

**Supplementary Table 3B**

**Current/Never (C/N) comparison in Non-Tumor (NT) lung tissues: down-regulated probes**

| **Probe ID** | **Fold-change < 0.6667** | **CN p-value < 0.001** | **Gene Symbol** | **Chromosomal Location** |
| --- | --- | --- | --- | --- |
| 213456_at | 0.2443 | 0.0000 | SOSTDC1 | 7p21.1 |
| 205347_s_at | 0.3913 | 0.0001 | TMSL8 | Xq21.33-q22.3 |
| 205433_at | 0.4488 | 0.0000 | BCHE | 3q26.1-q26.2 |
| 205898_at | 0.4633 | 0.0003 | CX3CR1 | 3p21|3p21.3 |
| 213316_at | 0.4712 | 0.0000 | KIAA1462 | 10p11.23 |
| 204731_at | 0.4779 | 0.0001 | TGFBR3 | 1p33-p32 |
| 213417_at | 0.4900 | 0.0002 | TBX2 | 17q23 |
| 213071_at | 0.4953 | 0.0000 | DPT | 1q12-q23 |
| 209220_at | 0.5051 | 0.0005 | GPC3 | Xq26.1 |
| 211276_at | 0.5066 | 0.0000 | TCEAL2 | Xq22.1-q22.3 |
| 204468_s_at | 0.5340 | 0.0001 | TIE1 | 1p34-p33 |
| 38241_at | 0.5428 | 0.0005 | BTN3A3 | 6p21.3 |
| 218736_s_at | 0.5485 | 0.0001 | PALMD | 1p22-p21 |
| 202524_s_at | 0.5563 | 0.0002 | SPOCK2 | 10pter-q25.3 |
| 219436_s_at | 0.5563 | 0.0006 | EMCN | 4q24 |
| 201641_at | 0.5601 | 0.0006 | BST2 | 19p13.2 |
| 203934_at | 0.5625 | 0.0003 | KDR | 4q11-q12 |
| 205498_at | 0.5685 | 0.0002 | GHR | 5p13-p12 |
| 203865_s_at | 0.5709 | 0.0003 | ADARB1 | 21q22.3 |
| 204677_at | 0.5720 | 0.0006 | CDH5 | 16q22.1 |
| 205528_s_at | 0.5737 | 0.0007 | RUNX1T1 | 8q22 |
| 218574_s_at | 0.5769 | 0.0001 | LMCD1 | 3p26-p24 |
| 213169_at | 0.5781 | 0.0001 | NA |  |
| 203349_s_at | 0.5812 | 0.0001 | ETV5 | 3q28 |
| 213032_at | 0.5829 | 0.0002 | NFIB | 9p24.1 |
| 205495_s_at | 0.5862 | 0.0009 | GNLY | 2p12-q11 |
| 202746_at | 0.5888 | 0.0000 | ITM2A | Xq13.3-Xq21.2 |
| 218804_at | 0.5902 | 0.0001 | TMEM16A | 11q13.3 |
| 213605_s_at | 0.5906 | 0.0003 | FLJ40092 | 5q13.2 |
| 204797_s_at | 0.5981 | 0.0002 | EML1 | 14q32 |
| 219315_s_at | 0.5981 | 0.0006 | C16orf30 | 16p13.3 |
| 212372_at | 0.5992 | 0.0004 | MYH10 | 17p13 |
| 203910_at | 0.6003 | 0.0007 | ARHGAP29 | 1p22.1 |
| 206595_at | 0.6004 | 0.0004 | CST6 | 11q13 |
| 208096_s_at | 0.6006 | 0.0002 | COL21A1 | 6p12.3-p11.2 |
| 202411_at | 0.6008 | 0.0007 | IFI27 | 14q32 |
| 204929_s_at | 0.6075 | 0.0003 | VAMP5 | 2p11.2 |
| 209656_s_at | 0.6092 | 0.0004 | TMEM47 | Xp11.4 |
| 203065_s_at | 0.6134 | 0.0001 | CAV1 | 7q31.1 |
| 202908_at | 0.6136 | 0.0001 | WFS1 | 4p16 |
| 219167_at | 0.6168 | 0.0000 | RASL12 | 15q11.2-q22.33 |
| 212822_at | 0.6177 | 0.0005 | HEG1 | 3q21.2 |
| 204821_at | 0.6210 | 0.0001 | BTN3A3 | 6p21.3 |
| 212609_s_at | 0.6241 | 0.0001 | AKT3 | 1q43-q44 |
| 220936_s_at | 0.6277 | 0.0004 | H2AFJ | 12p12 |
| 203071_at | 0.6280 | 0.0010 | SEMA3B | 3p21.3 |
| 204570_at | 0.6320 | 0.0004 | COX7A1 | 19q13.1 |
| 202177_at | 0.6333 | 0.0002 | GAS6 | 13q34 |
| 204428_s_at | 0.6352 | 0.0001 | LCAT | 16q22.1 |
| 218546_at | 0.6370 | 0.0003 | C1orf115 | 1q41 |
| 208789_at | 0.6372 | 0.0001 | PTRF | 17q21.2 |
| 218625_at | 0.6378 | 0.0001 | NRN1 | 6p25.1 |
| 201416_at | 0.6383 | 0.0003 | SOX4 | 6p22.3 |
| 203288_at | 0.6390 | 0.0005 | KIAA0355 | 19q13.11 |
| 205109_s_at | 0.6393 | 0.0001 | ARHGEF4 | 2q22 |
| 202478_at | 0.6408 | 0.0000 | TRIB2 | 2p24.3 |
| 218418_s_at | 0.6417 | 0.0001 | ANKRD25 | 19p13.2 |
| 209785_s_at | 0.6432 | 0.0007 | PLA2G4C | 19q13.3 |
| 214724_at | 0.6435 | 0.0009 | DIXDC1 |  |
| 213364_s_at | 0.6460 | 0.0008 | SNX1 | 15q22.31 |
| 219440_at | 0.6480 | 0.0007 | RAI2 | Xp22 |
| 201474_s_at | 0.6484 | 0.0000 | ITGA3 | 17q21.33 |
| 208634_s_at | 0.6485 | 0.0010 | MACF1 | 1p32-p31 |
| 203002_at | 0.6488 | 0.0001 | AMOTL2 | 3q21-q22 |
| 201150_s_at | 0.6488 | 0.0010 | TIMP3 | 22q12.1-q13.2|22q12.3 |
| 212914_at | 0.6525 | 0.0010 | CBX7 | 22q13.1 |
| 220351_at | 0.6546 | 0.0008 | CCRL1 | 3q22 |
| 202729_s_at | 0.6550 | 0.0007 | LTBP1 | 2p22-p21 |
| 204589_at | 0.6579 | 0.0003 | NUAK1 | 12q23.3 |
| 208760_at | 0.6592 | 0.0000 | UBE2I | 16p13.3 |
| 203688_at | 0.6592 | 0.0007 | PKD2 | 4q21-q23 |
| 201957_at | 0.6637 | 0.0006 | PPP1R12B | 1q32.1 |
| 215016_x_at | 0.6645 | 0.0002 | DST | 6p12-p11 |
| 203562_at | 0.6653 | 0.0010 | FEZ1 | 11q24.2 |
| 213675_at | 0.6657 | 0.0000 | ZDHHC3 | 3p21.31 |

**Supplementary Table 3C**

**Gene Ontology (GO) functional categories for the Current/Never (C/N) smoker comparison (up and down-regulated genes) in Non-Tumor (NT) lung tissues (p<0.01)**

| **GO ID** | **Total # genes classified in U133A** | **Down regulated** | **Up regulated** | **Change** | **p-value Up** | **p-value Down** | **p-value Changed** | **GO categories** |
| --- | --- | --- | --- | --- | --- | --- | --- | --- |
| 5578 | 215 | 6 | 2 | 8 | 0.0017 | 0.0943 | 0.0004 | extracellular matrix (sensu Metazoa) |
| 5576 | 897 | 10 | 8 | 18 | 0.0393 | 0.0009 | 0.0004 | extracellular region |
| 31012 | 218 | 6 | 2 | 8 | 0.0019 | 0.0966 | 0.0005 | extracellular matrix |
| 6968 | 90 | 2 | 3 | 5 | 0.1012 | 0.0013 | 0.0009 | cellular defense response |
| 7155 | 515 | 9 | 3 | 12 | 0.0034 | 0.1255 | 0.0012 | cell adhesion |
| 9605 | 448 | 4 | 7 | 11 | 0.2804 | 0.0001 | 0.0013 | response to external stimulus |
| 9653 | 539 | 9 | 3 | 12 | 0.0045 | 0.1387 | 0.0017 | morphogenesis |
| 7275 | 1561 | 19 | 5 | 24 | 0.0014 | 0.322 | 0.0019 | development |
| 6955 | 704 | 6 | 8 | 14 | 0.2445 | 0.0002 | 0.0021 | immune response |
| 9613 | 488 | 5 | 6 | 11 | 0.1671 | 0.0009 | 0.0025 | response to pest, pathogen or parasite |
| 51707 | 494 | 5 | 6 | 11 | 0.173 | 0.0009 | 0.0027 | response to other organism |
| 9611 | 351 | 3 | 6 | 9 | 0.3515 | 0.0001 | 0.0027 | response to wounding |
| 5509 | 654 | 9 | 4 | 13 | 0.0152 | 0.0694 | 0.003 | calcium ion binding |
| 44421 | 585 | 7 | 5 | 12 | 0.0594 | 0.0117 | 0.0034 | extracellular region part |
| 6952 | 781 | 6 | 8 | 14 | 0.3252 | 0.0003 | 0.0053 | defense response |
| 16020 | 3583 | 28 | 14 | 42 | 0.0548 | 0.0229 | 0.0057 | membrane |
| 7229 | 45 | 2 | 1 | 3 | 0.0296 | 0.1033 | 0.0063 | integrin-mediated signaling pathway |
| 9607 | 811 | 6 | 8 | 14 | 0.3578 | 0.0004 | 0.0074 | response to biotic stimulus |
| 1912 | 1 | 0 | 1 | 1 | 1 | 0.0024 | 0.0084 | positive regulation of immune cell mediated cytotoxicity |
| 4903 | 1 | 1 | 0 | 1 | 0.006 | 1 | 0.0084 | growth hormone receptor activity |
| 4502 | 1 | 0 | 1 | 1 | 1 | 0.0024 | 0.0084 | kynurenine 3-monooxygenase activity |
| 42640 | 1 | 0 | 1 | 1 | 1 | 0.0024 | 0.0084 | anagen |
| 47499 | 1 | 1 | 0 | 1 | 0.006 | 1 | 0.0084 | calcium-independent phospholipase A2 activity |
| 42599 | 1 | 0 | 1 | 1 | 1 | 0.0024 | 0.0084 | lamellar body |
| 4234 | 1 | 0 | 1 | 1 | 1 | 0.0024 | 0.0084 | macrophage elastase activity |
| 45954 | 1 | 0 | 1 | 1 | 1 | 0.0024 | 0.0084 | positive regulation of natural killer cell mediated cytotoxicity |
| 5932 | 1 | 1 | 0 | 1 | 0.006 | 1 | 0.0084 | basal body |
| 50783 | 1 | 1 | 0 | 1 | 0.006 | 1 | 0.0084 | cocaine metabolism |
| 31343 | 1 | 0 | 1 | 1 | 1 | 0.0024 | 0.0084 | positive regulation of cell killing |
| 9887 | 212 | 3 | 3 | 6 | 0.1338 | 0.014 | 0.0088 | organ morphogenesis |
| 48513 | 502 | 5 | 5 | 10 | 0.1811 | 0.0062 | 0.0092 | organ development |
| 51015 | 19 | 2 | 0 | 2 | 0.0057 | 1 | 0.0109 | actin filament binding |
| 4866 | 105 | 2 | 2 | 4 | 0.1305 | 0.0263 | 0.0117 | endopeptidase inhibitor activity |
| 50381 | 20 | 0 | 2 | 2 | 1 | 0.001 | 0.012 | unspecific monooxygenase activity |
| 30414 | 106 | 2 | 2 | 4 | 0.1325 | 0.0267 | 0.0121 | protease inhibitor activity |
| 5886 | 1523 | 14 | 7 | 21 | 0.0635 | 0.0636 | 0.0133 | plasma membrane |
| 6935 | 111 | 2 | 2 | 4 | 0.1427 | 0.0291 | 0.0141 | chemotaxis |
| 42330 | 111 | 2 | 2 | 4 | 0.1427 | 0.0291 | 0.0141 | taxis |
