# Supplementary material for: Gene Expression Signature of Cigarette Smoking and Its Role in Lung Adenocarcinoma Development and Survival
Source: PLoS One. 2008 Feb 20;3(2):e1651. doi: 10.1371/journal.pone.0001651 (PMC2249927; doi:10.1371/journal.pone.0001651)
Supplement: Appendix S4 — Comparison between Tumor (T) and Non-Tumor (NT) lung tissue for the genes whose expression significantly differentiates Current from Never smokers (C/N) in early stage lung Tumor (T). 4A C16orf30 and UBE21 transcription sites. 4B Comparison of C/N results in early stage Tumor (T) tissues vs. C/N results in Non-Tumor (NT) lung tissues by GSEA analysis. 4C Gene list from GSEA comparison of up-regulated C/N genes between early stage Tumor (T) tissues and Non-Tumor (NT) tissues. 4D Gene list from GSEA comparison of down-regulated C/N genes between early stage Tumor (T) tissues and Non-Tumor (NT) tissues. (0.51 MB DOC) [file pone.0001651.s004.doc]

**Appendix S4**

**Comparison between Tumor (T) and Non-Tumor (NT) lung tissue for the genes whose expression significantly differentiates Current from Never smokers (C/N) in early stage lung Tumor (T)**

**Supplementary Figure 4A**

**Description of analysis of C16orf30 and UBE21 loci, overlapping between C/N in T and C/N in NT (p-value≤0.001 and fold-change<0.6667)**

**Figure 4A legend**

We used the generic genome browser and data compiled at UCSC [1] to graphically evaluate transcriptional regulation, linkage disequlibrium and recombination at and between UBE2I and C16orf30, located in a gene-dense, transcriptionally active region on chromosome band 16p13.3. The two genes are transcribed on the + strand, where UBE2I is transcribed between base pairs 1,299,639-1,315,39 and C16orf30 is transcribed about 203 kbp downstream between 1,518,743 and 1,545,568, as shown in the genes track in blue. The sequences used to select probes for the Affymetrix HG-U133A chip are shown in the Affy U133 track in black. Both UBE2I and C16orf30 exhibit multiple 5’ and internal CpG islands,[2] shown in green; and conserved transcription factor binding sites (TFBS Conserved) and 5’ DNaseI hypersensitive sites (NHGRI DNaseI-HS),[3] shown in grey. Note that while both genes have conserved transcription factor sequence motifs, these sequence motif are not shared between the two genes. Note also that UBE2I, but not C16orf30, exhibits a 3’ miRNA sequence motif,[4] shown in green (T-ScanS miRNA). There is strong evidence of recombination, shown in grey, between the genes that peaks just upstream of C16orf30 in both the HapMap and Perlegen population samples, [5,6] and, accordingly, there is no significant pairwise linkage disequilibrium between the genes in the Caucasian HapMap population sample (LD CEU R↑2 track in red)

Reference List

1. Karolchik D, Baertsch R, Diekhans M, Furey TS, Hinrichs A, *et al.* (2003) The UCSC Genome Browser Database. Nucleic Acids Res 31: 51-54.

2. Gardiner-Garden M, Frommer M (1987) CpG islands in vertebrate genomes. J Mol Biol 196: 261-282.

3. Crawford GE, Holt IE, Mullikin JC, Tai D, Blakesley R, *et al.* (2004) Identifying gene regulatory elements by genome-wide recovery of DNase hypersensitive sites. Proc Natl Acad Sci U S A 101: 992-997.

4. Lewis BP, Burge CB, Bartel DP (2005) Conserved seed pairing, often flanked by adenosines, indicates that thousands of human genes are microRNA targets. Cell 120: 15-20.

5. The International HapMap Project. (2003) Nature 426: 789-796.

6. Hinds DA, Stuve LL, Nilsen GB, Halperin E, Eskin E, *et al.* (2005) Whole-genome patterns of common DNA variation in three human populations. Science 307: 1072-1079.

**Supplementary Figure 4A**

**Supplementary Figure 4B**

**Comparison of C/N results in early stage Tumor (T) tissues vs. C/N results in Non-Tumor (NT) lung tissues by GSEA analysis**

**Legend to Figure 4B**

**Left**: Running Enrichment Score (y axis) is calculated by walking down the entire list of probes from Affymetrix HG-U133A chip (numbered from 1 to 22,283 in the x axis) ordered by the ANOVA coefficients divided by the standard error values from the C/N comparison in NT. This running-sum statistic increases when a given probe is in the C/N in T Gene Set of interest and decreases when the probe is not in the C/N in T Gene Set, with the magnitude of increment depending on the strength of the correlation between the probe and the C/N comparison in NT. The Enrichment Score (ES) is the maximum deviation of the Running Enrichment Score from zero encountered in the random walk and reflects the degree to which the Gene Set is overrepresented at the extremes (top or bottom) of the entire ranked probe list. We report results for two different C/N in T Gene Sets: on the top, the 98 down-regulated probes, with ES=-0.62 and on the bottom, the 64 up-regulated probes, with ES=0.61. A leading edge subset of the Gene Set is defined as those probes in the Gene Set that appear in the probes ranked list at, or before, the point where the running sum reaches its maximum deviation from zero. The leading edge for the Gene Set of the C/N in T down-regulated probes contains 50 probes over 98 and the leading edge for the Gene Set of up-regulated probes contains 39 over 64 probes.

**Right**: distributions of ES values created using a permutation procedure for (top) the Gene Set of down-regulated probes in C/N in T and (bottom) the Gene Set of up-regulated probes in C/N in T. These distributions are used to calculate the statistical significance (nominal p-value) of the observed ES values (p-values 0.04 and 0.08).

**Supplementary Figure 4B**


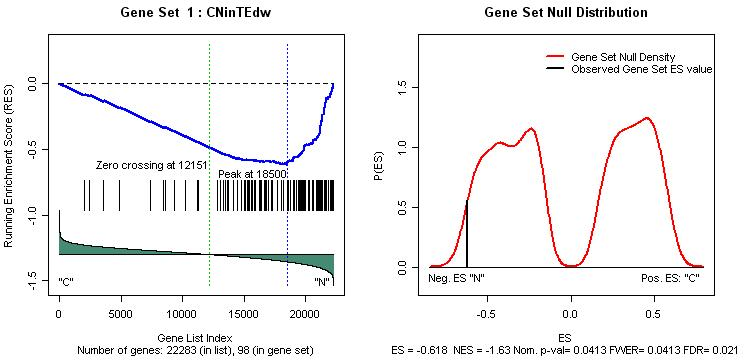


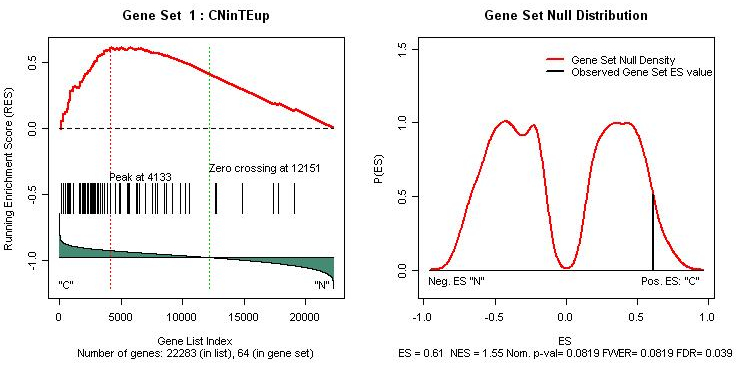


| **Gene Set from Tumor tissues data** | **# Probes in Gene Set** | **# Probes in Leading Edge** | **ES** | **p-value** |
| --- | --- | --- | --- | --- |
| CN down-regulated | 98 | 50 | -0.62 | 0.04 |
| CN up-regulated | 64 | 39 | 0.61 | 0.08 |

**Supplementary Table 4C**

**Gene list from GSEA comparison of up-regulated C/N genes between early stage Tumor (T) tissues and Non-Tumor (NT) tissues**

| **Probe ID** | **Gene Symbol** | **Core enrichment** | **GSEA index** |
| --- | --- | --- | --- |
| 212789_at | hCAP-D3 | YES | 1 |
| 203418_at | CCNA2 | YES | 2 |
| 220651_s_at | MCM10 | YES | 3 |
| 212290_at | SLC7A1 | YES | 4 |
| 212023_s_at | MKI67 | YES | 5 |
| 218355_at | KIF4A | YES | 6 |
| 209709_s_at | HMMR | YES | 7 |
| 206686_at | PDK1 | YES | 8 |
| 201761_at | MTHFD2 | YES | 9 |
| 210052_s_at | TPX2 | YES | 10 |
| 219306_at | KIF15 | YES | 11 |
| 204170_s_at | CKS2 | YES | 12 |
| 219918_s_at | ASPM | YES | 13 |
| 214007_s_at | PTK9 | YES | 14 |
| 204887_s_at | PLK4 | YES | 15 |
| 202095_s_at | BIRC5 | YES | 16 |
| 201292_at | TOP2A | YES | 17 |
| 211519_s_at | KIF2C | YES | 18 |
| 220295_x_at | DEPDC1 | YES | 19 |
| 218542_at | C10orf3 | YES | 20 |
| 204092_s_at | STK6 | YES | 21 |
| 207828_s_at | CENPF | YES | 22 |
| 219787_s_at | ECT2 | YES | 23 |
| 218662_s_at | HCAP-G | YES | 24 |
| 209642_at | BUB1 | YES | 25 |
| 212020_s_at | MKI67 | YES | 26 |
| 204822_at | TTK | YES | 27 |
| 209753_s_at | TMPO | YES | 28 |
| 218755_at | KIF20A | YES | 29 |
| 209408_at | KIF2C | YES | 30 |
| 204127_at | RFC3 | YES | 31 |
| 204146_at | RAD51AP1 | YES | 32 |
| 210559_s_at | CDC2 | YES | 33 |
| 201291_s_at | TOP2A | YES | 34 |
| 201635_s_at | FXR1 | YES | 35 |
| 204641_at | NEK2 | YES | 36 |
| 218349_s_at | ZWILCH | YES | 37 |
| 204649_at | TROAP | YES | 38 |
| 211762_s_at | KPNA2 | YES | 39 |
| 203362_s_at | MAD2L1 | NO | 40 |
| 204962_s_at | CENPA | NO | 41 |
| 218252_at | CKAP2 | NO | 42 |
| 203560_at | GGH | NO | 43 |
| 213189_at | DKFZp667G2110 | NO | 44 |
| 204203_at | CEBPG | NO | 45 |
| 209172_s_at | CENPF | NO | 46 |
| 218009_s_at | PRC1 | NO | 47 |
| 222077_s_at | RACGAP1 | NO | 48 |
| 203214_x_at | CDC2 | NO | 49 |
| 208777_s_at | PSMD11 | NO | 50 |
| 211080_s_at | NEK2 | NO | 51 |
| 201088_at | KPNA2 | NO | 52 |
| 222039_at | LOC146909 | NO | 53 |
| 209257_s_at | CSPG6 | NO | 54 |
| 200841_s_at | EPRS | NO | 55 |
| 219004_s_at | C21orf45 | NO | 56 |
| 202580_x_at | FOXM1 | NO | 57 |
| 203016_s_at | SSX2IP | NO | 58 |
| 201606_s_at | PWP1 | NO | 59 |
| 201637_s_at | FXR1 | NO | 60 |
| 201897_s_at | CKS1B | NO | 61 |
| 203017_s_at | SSX2IP | NO | 62 |
| 201636_at | FXR1 | NO | 63 |
| 201848_s_at | BNIP3 | NO | 64 |

**Supplementary Table 4D**

**Gene list from GSEA comparison of down-regulated C/N genes between early stage Tumor (T) tissues and Non-Tumor (NT) tissues**

| **Probe ID** | **Gene Symbol** | **Core enrichment** | **GSEA index** |
| --- | --- | --- | --- |
| 208760_at | UBE2I | YES | 1 |
| 208634_s_at | MACF1 | YES | 2 |
| 212914_at | CBX7 | YES | 3 |
| 212071_s_at | SPTBN1 | YES | 4 |
| 209667_at | CES2 | YES | 5 |
| 219909_at | MMP28 | YES | 6 |
| 201061_s_at | STOM | YES | 7 |
| 200810_s_at | CIRBP | YES | 8 |
| 204862_s_at | NME3 | YES | 9 |
| 211998_at | H3F3B | YES | 10 |
| 218679_s_at | VPS28 | YES | 11 |
| 203571_s_at | C10orf116 | YES | 12 |
| 206170_at | ADRB2 | YES | 13 |
| 217798_at | CNOT2 | YES | 14 |
| 205717_x_at | PCDHGC3 | YES | 15 |
| 221756_at | MGC17330 | YES | 16 |
| 201286_at | SDC1 | YES | 17 |
| 214894_x_at | MACF1 | YES | 18 |
| 209513_s_at | HSDL2 | YES | 19 |
| 201581_at | TXNDC13 | YES | 20 |
| 209263_x_at | TSPAN4 | YES | 21 |
| 221519_at | FBXW4 | YES | 22 |
| 200621_at | CSRP1 | YES | 23 |
| 212589_at | RRAS2 | YES | 24 |
| 208704_x_at | APLP2 | YES | 25 |
| 218686_s_at | RHBDF1 | YES | 26 |
| 212473_s_at | MICAL2 | YES | 27 |
| 201655_s_at | HSPG2 | YES | 28 |
| 210674_s_at | PCDHA12 | YES | 29 |
| 208248_x_at | APLP2 | YES | 30 |
| 201809_s_at | ENG | YES | 31 |
| 215399_s_at | OS9 | YES | 32 |
| 201287_s_at | SDC1 | YES | 33 |
| 209292_at | ID4 | YES | 34 |
| 208891_at | DUSP6 | YES | 35 |
| 205200_at | CLEC3B | YES | 36 |
| 208703_s_at | APLP2 | YES | 37 |
| 209264_s_at | TSPAN4 | YES | 38 |
| 204306_s_at | CD151 | YES | 39 |
| 208873_s_at | C5orf18 | YES | 40 |
| 208893_s_at | DUSP6 | YES | 41 |
| 210844_x_at | CTNNA1 | YES | 42 |
| 221127_s_at | RIG | YES | 43 |
| 201341_at | ENC1 | YES | 44 |
| 204276_at | TK2 | YES | 45 |
| 212950_at | GPR116 | YES | 46 |
| 213880_at | LGR5 | YES | 47 |
| 208890_s_at | PLXNB2 | YES | 48 |
| 200714_x_at | OS9 | YES | 49 |
| 200675_at | CD81 | YES | 50 |
| 201331_s_at | STAT6 | NO | 51 |
| 205559_s_at | PCSK5 | NO | 52 |
| 208702_x_at | APLP2 | NO | 53 |
| 204802_at | RRAD | NO | 54 |
| 201651_s_at | PACSIN2 | NO | 55 |
| 212622_at | TMEM41B | NO | 56 |
| 203227_s_at | TSPAN31 | NO | 57 |
| 212472_at | MICAL2 | NO | 58 |
| 206528_at | TRPC6 | NO | 59 |
| 213244_at | SCAMP4 | NO | 60 |
| 212576_at | MGRN1 | NO | 61 |
| 212256_at | GALNT10 | NO | 62 |
| 201360_at | CST3 | NO | 63 |
| 204916_at | RAMP1 | NO | 64 |
| 202739_s_at | PHKB | NO | 65 |
| 211404_s_at | APLP2 | NO | 66 |
| 205539_at | AVIL | NO | 67 |
| 202071_at | SDC4 | NO | 68 |
| 200696_s_at | GSN | NO | 69 |
| 221489_s_at | SPRY4 | NO | 70 |
| 209499_x_at | TNFSF13 | NO | 71 |
| 217287_s_at | TRPC6 | NO | 72 |
| 204803_s_at | RRAD | NO | 73 |
| 219206_x_at | TMBIM4 | NO | 74 |
| 205931_s_at | CREB5 | NO | 75 |
| 210314_x_at | TNFSF13 | NO | 76 |
| 201282_at | OGDH | NO | 77 |
| 209373_at | MALL | NO | 78 |
| 200678_x_at | GRN | NO | 79 |
| 200972_at | TSPAN3 | NO | 80 |
| 210788_s_at | DHRS7 | NO | 81 |
| 200973_s_at | TSPAN3 | NO | 82 |
| 212334_at | GNS | NO | 83 |
| 212951_at | GPR116 | NO | 84 |
| 206114_at | EPHA4 | NO | 85 |
| 215684_s_at | ASCC2 | NO | 86 |
| 220622_at | LRRC31 | NO | 87 |
| 218368_s_at | TNFRSF12A | NO | 88 |
| 210507_s_at | AVIL | NO | 89 |
| 218211_s_at | MLPH | NO | 90 |
| 217967_s_at | C1orf24 | NO | 91 |
| 209605_at | TST | NO | 92 |
| 203226_s_at | TSPAN31 | NO | 93 |
| 202068_s_at | LDLR | NO | 94 |
| 200766_at | CTSD | NO | 95 |
| 203757_s_at | CEACAM6 | NO | 96 |
| 214841_at | CNIH3 | NO | 97 |
| 202284_s_at | CDKN1A | NO | 98 |
