# Supplementary material for: Gene Expression Signature of Cigarette Smoking and Its Role in Lung Adenocarcinoma Development and Survival
Source: PLoS One. 2008 Feb 20;3(2):e1651. doi: 10.1371/journal.pone.0001651 (PMC2249927; doi:10.1371/journal.pone.0001651)
Supplement: Appendix S5 — Mortality risk in smokers associated with the expression of genes differentiating Current from Never smokers (C/N) in Tumor and Non-Tumor tissue samples. 5A Current/Never (C/N) genes and related mortality risk in Tumor and Non-Tumor lung tissues (all stages) from Current and Former smokers. (0.55 MB DOC) [file pone.0001651.s005.doc]

**Appendix S5**

**Mortality risk in smokers associated with the expression of genes differentiating Current from Never smokers (C/N) in Tumor and Non-Tumor tissue samples**

**Supplementary Table 5A**

**Current/Never (C/N) smoking genes and related mortality risk in Tumor and Non-Tumor lung tissues (all stages) from Current (n=28) and Former (n=26) smokers.**

Model adjusted for smoking status, sex, and tumor stage.

| **Probe ID** | **Gene  Symbol** | **Non-Tumor RR** | **95% CI** | | **p value** |  | **Tumor RR** | **95% CI** | | **p value** |
| --- | --- | --- | --- | --- | --- | --- | --- | --- | --- | --- |
| 204641_at | NEK2 | 2.65 | 1.50 | 4.68 | 0.0008 |  | 1.16 | 0.72 | 1.88 | 0.5300 |
| 204822_at | TTK | 2.91 | 1.53 | 5.51 | 0.0011 |  | 1.32 | 0.78 | 2.24 | 0.3000 |
| 201292_at | TOP2A | 3.08 | 1.43 | 6.66 | 0.0041 |  | 0.76 | 0.49 | 1.16 | 0.2000 |
| 219306_at | KIF15 | 2.84 | 1.37 | 5.85 | 0.0048 |  | 0.92 | 0.61 | 1.39 | 0.7000 |
| 202068_s_at | LDLR | 0.48 | 0.29 | 0.82 | 0.0068 |  | 0.77 | 0.43 | 1.36 | 0.3600 |
| 218542_at | C10orf3 | 2.66 | 1.31 | 5.42 | 0.0068 |  | 0.89 | 0.55 | 1.44 | 0.6200 |
| 209642_at | BUB1 | 2.76 | 1.30 | 5.87 | 0.0084 |  | 0.84 | 0.53 | 1.30 | 0.4300 |
| 214894_x_at | MACF1 | 0.39 | 0.19 | 0.79 | 0.0091 |  | 0.81 | 0.48 | 1.36 | 0.4300 |
| 218804_at | TMEM16A | 0.42 | 0.22 | 0.81 | 0.0095 |  | 1.12 | 0.75 | 1.67 | 0.5900 |
| 222077_s_at | RACGAP1 | 2.82 | 1.27 | 6.24 | 0.0110 |  | 0.92 | 0.60 | 1.41 | 0.7000 |
| 203362_s_at | MAD2L1 | 2.20 | 1.19 | 4.06 | 0.0120 |  | 1.01 | 0.65 | 1.58 | 0.9600 |
| 205749_at | CYP1A1 | 1.92 | 1.15 | 3.21 | 0.0130 |  | 0.83 | 0.54 | 1.28 | 0.3900 |
| 202095_s_at | BIRC5 | 2.38 | 1.18 | 4.81 | 0.0150 |  | 0.87 | 0.54 | 1.38 | 0.5400 |
| 218355_at | KIF4A | 2.40 | 1.19 | 4.87 | 0.0150 |  | 0.78 | 0.51 | 1.19 | 0.2500 |
| 204731_at | TGFBR3 | 0.52 | 0.30 | 0.90 | 0.0180 |  | 1.14 | 0.71 | 1.82 | 0.5900 |
| 205576_at | SERPIND1 | 2.84 | 1.17 | 6.86 | 0.0210 |  | 0.69 | 0.38 | 1.25 | 0.2200 |
| 200766_at | CTSD | 2.09 | 1.11 | 3.94 | 0.0220 |  | 0.80 | 0.54 | 1.19 | 0.2800 |
| 212789_at | hCAP-D3 | 2.10 | 1.11 | 3.96 | 0.0220 |  | 1.13 | 0.68 | 1.86 | 0.6400 |
| 201282_at | OGDH | 2.05 | 1.11 | 3.81 | 0.0230 |  | 1.18 | 0.72 | 1.94 | 0.5100 |
| 205539_at | AVIL | 0.59 | 0.38 | 0.93 | 0.0240 |  | 0.76 | 0.49 | 1.17 | 0.2200 |
| 219725_at | TREM2 | 2.20 | 1.11 | 4.37 | 0.0240 |  | 0.83 | 0.50 | 1.37 | 0.4700 |
| 212822_at | HEG1 | 0.46 | 0.24 | 0.91 | 0.0250 |  | 1.01 | 0.61 | 1.66 | 0.9800 |
| 220380_at | DNASE2B | 2.50 | 1.10 | 5.64 | 0.0280 |  | 0.80 | 0.52 | 1.23 | 0.3100 |
| 204580_at | MMP12 | 2.11 | 1.08 | 4.12 | 0.0290 |  | 0.69 | 0.44 | 1.10 | 0.1200 |
| 219440_at | RAI2 | 0.50 | 0.27 | 0.93 | 0.0290 |  | 1.08 | 0.68 | 1.71 | 0.7600 |
| 209172_s_at | CENPF | 2.19 | 1.08 | 4.44 | 0.0300 |  | 1.19 | 0.74 | 1.91 | 0.4600 |
| 202437_s_at | CYP1B1 | 2.72 | 1.09 | 6.81 | 0.0320 |  | 0.96 | 0.62 | 1.50 | 0.8600 |
| 208702_x_at | APLP2 | 0.54 | 0.31 | 0.95 | 0.0320 |  | 0.73 | 0.50 | 1.07 | 0.1100 |
| 219787_s_at | ECT2 | 1.95 | 1.06 | 3.60 | 0.0320 |  | 1.15 | 0.73 | 1.83 | 0.5400 |
| 206420_at | IGSF6 | 1.98 | 1.06 | 3.71 | 0.0330 |  | 0.66 | 0.40 | 1.10 | 0.1100 |
| 210052_s_at | TPX2 | 2.07 | 1.06 | 4.03 | 0.0330 |  | 0.83 | 0.56 | 1.25 | 0.3800 |
| 201416_at | SOX4 | 1.99 | 1.06 | 3.75 | 0.0340 |  | 0.85 | 0.52 | 1.38 | 0.5100 |
| 209709_s_at | HMMR | 1.96 | 1.05 | 3.64 | 0.0340 |  | 0.96 | 0.65 | 1.41 | 0.8200 |
| 200714_x_at | OS9 | 1.67 | 1.03 | 2.71 | 0.0360 |  | 0.78 | 0.50 | 1.24 | 0.2900 |
| 219918_s_at | ASPM | 2.01 | 1.04 | 3.92 | 0.0390 |  | 0.97 | 0.61 | 1.54 | 0.9100 |
| 212020_s_at | MKI67 | 1.63 | 1.02 | 2.59 | 0.0400 |  | 0.71 | 0.44 | 1.14 | 0.1500 |
| 214007_s_at | PTK9 | 0.57 | 0.34 | 0.98 | 0.0400 |  | 1.22 | 0.79 | 1.90 | 0.3700 |
| 214841_at | CNIH3 | 1.88 | 1.03 | 3.45 | 0.0400 |  | 1.00 | 0.68 | 1.46 | 0.9900 |
| 212334_at | GNS | 1.85 | 1.02 | 3.36 | 0.0430 |  | 1.24 | 0.75 | 2.04 | 0.4000 |
| 201291_s_at | TOP2A | 1.92 | 1.01 | 3.62 | 0.0450 |  | 0.86 | 0.58 | 1.27 | 0.4400 |
| 202580_x_at | FOXM1 | 1.86 | 1.01 | 3.41 | 0.0450 |  | 1.02 | 0.66 | 1.58 | 0.9300 |
| 208893_s_at | DUSP6 | 0.60 | 0.36 | 0.99 | 0.0460 |  | 0.88 | 0.54 | 1.41 | 0.5800 |
| 202284_s_at | CDKN1A | 0.57 | 0.33 | 1.00 | 0.0480 |  | 1.09 | 0.69 | 1.70 | 0.7100 |
| 204962_s_at | CENPA | 1.81 | 1.00 | 3.28 | 0.0480 |  | 0.90 | 0.59 | 1.39 | 0.6300 |
| 208890_s_at | PLXNB2 | 1.82 | 1.01 | 3.29 | 0.0480 |  | 0.83 | 0.53 | 1.30 | 0.4200 |
| 202435_s_at | CYP1B1 | 1.97 | 1.00 | 3.88 | 0.0510 |  | 1.04 | 0.66 | 1.62 | 0.8800 |
| 218252_at | CKAP2 | 1.86 | 0.99 | 3.50 | 0.0540 |  | 0.86 | 0.54 | 1.37 | 0.5200 |
| 205433_at | BCHE | 0.53 | 0.28 | 1.01 | 0.0550 |  | 0.95 | 0.52 | 1.74 | 0.8700 |
| 219436_s_at | EMCN | 0.56 | 0.30 | 1.03 | 0.0640 |  | 1.39 | 0.85 | 2.27 | 0.1900 |
| 205738_s_at | FABP3 | 1.54 | 0.97 | 2.43 | 0.0660 |  | 1.33 | 0.88 | 1.99 | 0.1700 |
| 212609_s_at | AKT3 | 0.60 | 0.34 | 1.04 | 0.0690 |  | 1.13 | 0.72 | 1.77 | 0.6100 |
| 201341_at | ENC1 | 1.71 | 0.96 | 3.04 | 0.0710 |  | 1.05 | 0.68 | 1.63 | 0.8200 |
| 210184_at | ITGAX | 1.83 | 0.95 | 3.52 | 0.0710 |  | 0.80 | 0.49 | 1.30 | 0.3600 |
| 201636_at | FXR1 | 1.66 | 0.95 | 2.88 | 0.0750 |  | 1.33 | 0.82 | 2.17 | 0.2500 |
| 204203_at | CEBPG | 0.60 | 0.34 | 1.05 | 0.0750 |  | 0.91 | 0.58 | 1.45 | 0.7000 |
| 210133_at | CCL11 | 0.60 | 0.34 | 1.05 | 0.0750 |  | 0.87 | 0.57 | 1.32 | 0.5200 |
| 206170_at | ADRB2 | 0.65 | 0.40 | 1.05 | 0.0770 |  | 0.79 | 0.46 | 1.33 | 0.3700 |
| 213880_at | LGR5 | 0.55 | 0.28 | 1.07 | 0.0790 |  | 1.59 | 1.10 | 2.30 | 0.0140 |
| 209292_at | ID4 | 0.60 | 0.34 | 1.06 | 0.0810 |  | 1.23 | 0.78 | 1.93 | 0.3700 |
| 221127_s_at | RIG | 0.63 | 0.37 | 1.06 | 0.0810 |  | 0.87 | 0.54 | 1.42 | 0.5800 |
| 201641_at | BST2 | 1.72 | 0.93 | 3.16 | 0.0820 |  | 0.84 | 0.55 | 1.29 | 0.4300 |
| 213244_at | SCAMP4 | 1.76 | 0.92 | 3.39 | 0.0880 |  | 1.22 | 0.75 | 2.00 | 0.4200 |
| 201637_s_at | FXR1 | 1.80 | 0.91 | 3.55 | 0.0910 |  | 2.78 | 1.54 | 5.03 | 0.0007 |
| 220428_at | CD207 | 1.94 | 0.90 | 4.19 | 0.0910 |  | 0.79 | 0.51 | 1.22 | 0.2900 |
| 206528_at | TRPC6 | 1.57 | 0.93 | 2.64 | 0.0930 |  | 1.24 | 0.79 | 1.94 | 0.3500 |
| 203065_s_at | CAV1 | 0.60 | 0.32 | 1.10 | 0.0990 |  | 1.12 | 0.71 | 1.78 | 0.6200 |
| 209753_s_at | TMPO | 0.66 | 0.40 | 1.09 | 0.1000 |  | 0.76 | 0.49 | 1.17 | 0.2100 |
| 213071_at | DPT | 0.46 | 0.18 | 1.17 | 0.1000 |  | 0.88 | 0.56 | 1.40 | 0.6000 |
| 203227_s_at | TSPAN31 | 1.49 | 0.92 | 2.42 | 0.1100 |  | 0.98 | 0.67 | 1.44 | 0.9100 |
| 211404_s_at | APLP2 | 0.64 | 0.37 | 1.10 | 0.1100 |  | 0.76 | 0.52 | 1.12 | 0.1700 |
| 211998_at | H3F3B | 0.66 | 0.39 | 1.11 | 0.1100 |  | 1.17 | 0.76 | 1.80 | 0.4800 |
| 212372_at | MYH10 | 0.62 | 0.35 | 1.12 | 0.1100 |  | 0.99 | 0.61 | 1.61 | 0.9700 |
| 218662_s_at | HCAP-G | 1.69 | 0.89 | 3.18 | 0.1100 |  | 1.33 | 0.82 | 2.16 | 0.2500 |
| 200972_at | TSPAN3 | 1.51 | 0.90 | 2.53 | 0.1200 |  | 0.80 | 0.49 | 1.30 | 0.3700 |
| 202436_s_at | CYP1B1 | 1.80 | 0.87 | 3.75 | 0.1200 |  | 1.00 | 0.65 | 1.55 | 1.0000 |
| 204170_s_at | CKS2 | 1.70 | 0.87 | 3.33 | 0.1200 |  | 1.12 | 0.73 | 1.71 | 0.5900 |
| 208634_s_at | MACF1 | 0.67 | 0.40 | 1.11 | 0.1200 |  | 0.81 | 0.53 | 1.23 | 0.3200 |
| 218755_at | KIF20A | 1.71 | 0.86 | 3.37 | 0.1200 |  | 1.23 | 0.73 | 2.08 | 0.4400 |
| 221489_s_at | SPRY4 | 0.65 | 0.38 | 1.12 | 0.1200 |  | 1.15 | 0.74 | 1.79 | 0.5400 |
| 200678_x_at | GRN | 1.59 | 0.87 | 2.89 | 0.1300 |  | 0.66 | 0.43 | 1.00 | 0.0510 |
| 202524_s_at | SPOCK2 | 0.70 | 0.45 | 1.11 | 0.1300 |  | 1.04 | 0.68 | 1.59 | 0.8500 |
| 208248_x_at | APLP2 | 1.53 | 0.89 | 2.63 | 0.1300 |  | 0.82 | 0.55 | 1.23 | 0.3300 |
| 220625_s_at | ELF5 | 1.65 | 0.86 | 3.20 | 0.1300 |  | 1.18 | 0.75 | 1.85 | 0.4700 |
| 222039_at | LOC146909 | 1.87 | 0.83 | 4.19 | 0.1300 |  | 1.04 | 0.66 | 1.62 | 0.8800 |
| 205185_at | SPINK5 | 1.70 | 0.84 | 3.44 | 0.1400 |  | 0.94 | 0.65 | 1.38 | 0.7700 |
| 203560_at | GGH | 1.53 | 0.86 | 2.71 | 0.1500 |  | 1.61 | 0.86 | 3.03 | 0.1400 |
| 207828_s_at | CENPF | 1.63 | 0.84 | 3.17 | 0.1500 |  | 1.12 | 0.69 | 1.83 | 0.6400 |
| 213456_at | SOSTDC1 | 0.64 | 0.35 | 1.17 | 0.1500 |  | 1.25 | 0.79 | 1.97 | 0.3400 |
| 220651_s_at | MCM10 | 1.59 | 0.84 | 3.02 | 0.1500 |  | 0.95 | 0.56 | 1.63 | 0.8700 |
| 212914_at | CBX7 | 0.67 | 0.39 | 1.18 | 0.1600 |  | 0.73 | 0.43 | 1.23 | 0.2400 |
| 220936_s_at | H2AFJ | 1.40 | 0.88 | 2.22 | 0.1600 |  | 1.10 | 0.70 | 1.72 | 0.6800 |
| 202478_at | TRIB2 | 1.61 | 0.82 | 3.15 | 0.1700 |  | 1.22 | 0.82 | 1.82 | 0.3300 |
| 204916_at | RAMP1 | 1.36 | 0.88 | 2.10 | 0.1700 |  | 1.04 | 0.65 | 1.66 | 0.8800 |
| 215399_s_at | OS9 | 1.58 | 0.82 | 3.05 | 0.1700 |  | 0.65 | 0.39 | 1.07 | 0.0910 |
| 201360_at | CST3 | 1.54 | 0.81 | 2.93 | 0.1800 |  | 0.80 | 0.49 | 1.31 | 0.3700 |
| 204092_s_at | STK6 | 1.48 | 0.84 | 2.61 | 0.1800 |  | 0.88 | 0.55 | 1.41 | 0.5900 |
| 209373_at | MALL | 1.49 | 0.83 | 2.68 | 0.1800 |  | 0.86 | 0.53 | 1.40 | 0.5400 |
| 213695_at | PON3 | 1.77 | 0.77 | 4.09 | 0.1800 |  | 0.97 | 0.63 | 1.49 | 0.8800 |
| 200810_s_at | CIRBP | 0.69 | 0.40 | 1.21 | 0.1900 |  | 1.06 | 0.65 | 1.75 | 0.8100 |
| 201331_s_at | STAT6 | 1.38 | 0.85 | 2.24 | 0.1900 |  | 1.11 | 0.76 | 1.64 | 0.5800 |
| 201651_s_at | PACSIN2 | 1.40 | 0.85 | 2.32 | 0.1900 |  | 0.41 | 0.22 | 0.76 | 0.0046 |
| 201957_at | PPP1R12B | 1.42 | 0.85 | 2.38 | 0.1900 |  | 0.99 | 0.58 | 1.68 | 0.9600 |
| 201581_at | TXNDC13 | 0.70 | 0.40 | 1.21 | 0.2000 |  | 0.66 | 0.37 | 1.16 | 0.1500 |
| 219837_s_at | CYTL1 | 0.58 | 0.25 | 1.33 | 0.2000 |  | 0.91 | 0.57 | 1.45 | 0.6800 |
| 201635_s_at | FXR1 | 0.74 | 0.45 | 1.19 | 0.2100 |  | 1.46 | 0.94 | 2.29 | 0.0950 |
| 203865_s_at | ADARB1 | 0.66 | 0.35 | 1.26 | 0.2100 |  | 1.27 | 0.78 | 2.05 | 0.3300 |
| 201088_at | KPNA2 | 1.41 | 0.81 | 2.45 | 0.2200 |  | 0.88 | 0.54 | 1.44 | 0.6200 |
| 203226_s_at | TSPAN31 | 0.72 | 0.42 | 1.22 | 0.2200 |  | 0.79 | 0.51 | 1.20 | 0.2700 |
| 208760_at | UBE2I | 0.71 | 0.41 | 1.23 | 0.2200 |  | 0.88 | 0.56 | 1.38 | 0.5800 |
| 208891_at | DUSP6 | 0.73 | 0.45 | 1.20 | 0.2200 |  | 1.01 | 0.60 | 1.71 | 0.9700 |
| 209513_s_at | HSDL2 | 0.68 | 0.37 | 1.25 | 0.2200 |  | 0.94 | 0.59 | 1.49 | 0.7800 |
| 206686_at | PDK1 | 0.74 | 0.46 | 1.20 | 0.2300 |  | 1.04 | 0.68 | 1.57 | 0.8700 |
| 212472_at | MICAL2 | 1.38 | 0.81 | 2.33 | 0.2300 |  | 0.69 | 0.44 | 1.08 | 0.1000 |
| 218546_at | C1orf115 | 0.70 | 0.39 | 1.25 | 0.2300 |  | 1.44 | 0.88 | 2.37 | 0.1500 |
| 212951_at | GPR116 | 1.42 | 0.79 | 2.53 | 0.2400 |  | 0.92 | 0.60 | 1.40 | 0.6900 |
| 213032_at | NFIB | 1.40 | 0.80 | 2.47 | 0.2400 |  | 0.99 | 0.54 | 1.83 | 0.9800 |
| 200841_s_at | EPRS | 0.75 | 0.46 | 1.22 | 0.2500 |  | 1.49 | 0.88 | 2.50 | 0.1300 |
| 203934_at | KDR | 0.75 | 0.46 | 1.23 | 0.2500 |  | 0.81 | 0.53 | 1.24 | 0.3400 |
| 201761_at | MTHFD2 | 1.41 | 0.78 | 2.58 | 0.2600 |  | 0.93 | 0.58 | 1.46 | 0.7400 |
| 203418_at | CCNA2 | 1.41 | 0.75 | 2.66 | 0.2800 |  | 0.88 | 0.58 | 1.35 | 0.5600 |
| 212023_s_at | MKI67 | 1.27 | 0.82 | 1.97 | 0.2900 |  | 1.10 | 0.69 | 1.76 | 0.7000 |
| 204802_at | RRAD | 0.75 | 0.43 | 1.30 | 0.3000 |  | 0.88 | 0.55 | 1.40 | 0.5900 |
| 212473_s_at | MICAL2 | 1.41 | 0.74 | 2.69 | 0.3000 |  | 0.80 | 0.51 | 1.26 | 0.3400 |
| 212950_at | GPR116 | 0.78 | 0.49 | 1.24 | 0.3000 |  | 0.88 | 0.57 | 1.35 | 0.5500 |
| 218211_s_at | MLPH | 0.76 | 0.44 | 1.29 | 0.3100 |  | 0.68 | 0.42 | 1.11 | 0.1200 |
| 201150_s_at | TIMP3 | 0.73 | 0.40 | 1.35 | 0.3200 |  | 0.79 | 0.51 | 1.22 | 0.2900 |
| 203016_s_at | SSX2IP | 1.27 | 0.80 | 2.03 | 0.3200 |  | 0.99 | 0.62 | 1.57 | 0.9500 |
| 204589_at | NUAK1 | 1.35 | 0.74 | 2.47 | 0.3200 |  | 0.85 | 0.56 | 1.29 | 0.4300 |
| 215016_x_at | DST | 0.75 | 0.43 | 1.32 | 0.3200 |  | 0.84 | 0.53 | 1.32 | 0.4400 |
| 203288_at | KIAA0355 | 0.79 | 0.49 | 1.27 | 0.3300 |  | 0.79 | 0.47 | 1.32 | 0.3700 |
| 204797_s_at | EML1 | 0.74 | 0.40 | 1.36 | 0.3300 |  | 1.46 | 0.94 | 2.28 | 0.0940 |
| 204803_s_at | RRAD | 0.76 | 0.44 | 1.32 | 0.3300 |  | 0.96 | 0.60 | 1.53 | 0.8700 |
| 210314_x_at | TNFSF13 | 1.33 | 0.75 | 2.37 | 0.3300 |  | 0.99 | 0.67 | 1.47 | 0.9500 |
| 219315_s_at | C16orf30 | 0.74 | 0.40 | 1.36 | 0.3300 |  | 0.89 | 0.56 | 1.41 | 0.6200 |
| 221756_at | MGC17330 | 0.73 | 0.38 | 1.39 | 0.3300 |  | 0.95 | 0.58 | 1.56 | 0.8500 |
| 204468_s_at | TIE1 | 1.34 | 0.74 | 2.42 | 0.3400 |  | 1.35 | 0.88 | 2.07 | 0.1700 |
| 208704_x_at | APLP2 | 1.27 | 0.78 | 2.05 | 0.3400 |  | 0.86 | 0.58 | 1.30 | 0.4800 |
| 218679_s_at | VPS28 | 1.31 | 0.75 | 2.28 | 0.3400 |  | 1.12 | 0.74 | 1.70 | 0.5800 |
| 200675_at | CD81 | 1.41 | 0.69 | 2.91 | 0.3500 |  | 0.88 | 0.60 | 1.30 | 0.5200 |
| 214724_at | DIXDC1 | 1.24 | 0.79 | 1.95 | 0.3500 |  | 0.92 | 0.60 | 1.43 | 0.7200 |
| 218574_s_at | LMCD1 | 0.75 | 0.42 | 1.36 | 0.3500 |  | 1.17 | 0.79 | 1.72 | 0.4300 |
| 200696_s_at | GSN | 1.31 | 0.73 | 2.36 | 0.3600 |  | 0.54 | 0.31 | 0.92 | 0.0230 |
| 203910_at | ARHGAP29 | 0.79 | 0.47 | 1.31 | 0.3600 |  | 0.70 | 0.43 | 1.15 | 0.1600 |
| 205717_x_at | PCDHGC3 | 1.37 | 0.70 | 2.69 | 0.3600 |  | 1.30 | 0.77 | 2.18 | 0.3200 |
| 213605_s_at | FLJ40092 | 1.26 | 0.77 | 2.05 | 0.3600 |  | 0.93 | 0.61 | 1.42 | 0.7300 |
| 201897_s_at | CKS1B | 1.26 | 0.76 | 2.07 | 0.3700 |  | 0.96 | 0.61 | 1.50 | 0.8500 |
| 205528_s_at | RUNX1T1 | 1.26 | 0.76 | 2.10 | 0.3700 |  | 0.91 | 0.58 | 1.42 | 0.6700 |
| 213675_at | ZDHHC3 | 1.32 | 0.72 | 2.44 | 0.3700 |  | 1.14 | 0.75 | 1.76 | 0.5400 |
| 220622_at | LRRC31 | 0.79 | 0.48 | 1.31 | 0.3700 |  | 0.72 | 0.37 | 1.36 | 0.3100 |
| 213364_s_at | SNX1 | 0.78 | 0.45 | 1.36 | 0.3800 |  | 1.04 | 0.70 | 1.53 | 0.8600 |
| 218418_s_at | ANKRD25 | 0.77 | 0.43 | 1.38 | 0.3800 |  | 1.02 | 0.66 | 1.57 | 0.9300 |
| 204887_s_at | PLK4 | 1.32 | 0.69 | 2.50 | 0.4000 |  | 0.83 | 0.55 | 1.28 | 0.4000 |
| 210559_s_at | CDC2 | 1.32 | 0.67 | 2.60 | 0.4200 |  | 0.94 | 0.60 | 1.46 | 0.7900 |
| 220295_x_at | DEPDC1 | 1.28 | 0.71 | 2.30 | 0.4200 |  | 0.92 | 0.56 | 1.52 | 0.7500 |
| 203214_x_at | CDC2 | 1.26 | 0.71 | 2.23 | 0.4300 |  | 0.87 | 0.56 | 1.37 | 0.5600 |
| 206114_at | EPHA4 | 1.23 | 0.73 | 2.07 | 0.4300 |  | 0.74 | 0.45 | 1.22 | 0.2400 |
| 209605_at | TST | 1.30 | 0.67 | 2.51 | 0.4400 |  | 0.97 | 0.64 | 1.48 | 0.9000 |
| 210788_s_at | DHRS7 | 0.82 | 0.49 | 1.37 | 0.4400 |  | 0.72 | 0.46 | 1.13 | 0.1500 |
| 213189_at | DKFZp667G2110 | 0.81 | 0.48 | 1.37 | 0.4400 |  | 2.00 | 1.19 | 3.36 | 0.0088 |
| 218368_s_at | TNFRSF12A | 0.81 | 0.47 | 1.39 | 0.4400 |  | 1.00 | 0.64 | 1.57 | 0.9900 |
| 221266_s_at | TM7SF4 | 1.39 | 0.60 | 3.21 | 0.4400 |  | 0.90 | 0.59 | 1.37 | 0.6300 |
| 218625_at | NRN1 | 0.83 | 0.51 | 1.35 | 0.4500 |  | 0.69 | 0.41 | 1.16 | 0.1600 |
| 202908_at | WFS1 | 0.82 | 0.48 | 1.39 | 0.4600 |  | 0.99 | 0.67 | 1.49 | 0.9800 |
| 205109_s_at | ARHGEF4 | 0.80 | 0.43 | 1.46 | 0.4600 |  | 1.69 | 1.02 | 2.79 | 0.0400 |
| 209656_s_at | TMEM47 | 0.83 | 0.51 | 1.36 | 0.4600 |  | 0.99 | 0.68 | 1.44 | 0.9500 |
| 210674_s_at | PCDHA12 | 0.82 | 0.47 | 1.41 | 0.4700 |  | 1.01 | 0.62 | 1.66 | 0.9600 |
| 208096_s_at | COL21A1 | 1.23 | 0.69 | 2.19 | 0.4800 |  | 0.58 | 0.33 | 1.02 | 0.0600 |
| 212576_at | MGRN1 | 1.17 | 0.73 | 1.88 | 0.5000 |  | 1.06 | 0.66 | 1.71 | 0.8000 |
| 219909_at | MMP28 | 0.84 | 0.50 | 1.40 | 0.5000 |  | 0.87 | 0.52 | 1.47 | 0.6000 |
| 202739_s_at | PHKB | 1.20 | 0.70 | 2.04 | 0.5100 |  | 0.94 | 0.59 | 1.49 | 0.7900 |
| 219612_s_at | FGG | 0.82 | 0.46 | 1.48 | 0.5100 |  | 1.72 | 1.04 | 2.84 | 0.0350 |
| 203349_s_at | ETV5 | 1.20 | 0.69 | 2.08 | 0.5200 |  | 1.10 | 0.69 | 1.78 | 0.6800 |
| 204127_at | RFC3 | 1.18 | 0.71 | 1.96 | 0.5200 |  | 0.92 | 0.56 | 1.52 | 0.7500 |
| 204276_at | TK2 | 1.20 | 0.68 | 2.11 | 0.5200 |  | 0.81 | 0.48 | 1.34 | 0.4000 |
| 208789_at | PTRF | 1.19 | 0.70 | 2.01 | 0.5200 |  | 1.01 | 0.65 | 1.56 | 0.9800 |
| 204677_at | CDH5 | 0.85 | 0.52 | 1.39 | 0.5300 |  | 1.03 | 0.68 | 1.55 | 0.8900 |
| 217967_s_at | C1orf24 | 1.15 | 0.74 | 1.79 | 0.5300 |  | 0.79 | 0.52 | 1.22 | 0.2900 |
| 203922_s_at | CYBB | 1.24 | 0.63 | 2.44 | 0.5400 |  | 0.66 | 0.41 | 1.05 | 0.0790 |
| 204146_at | RAD51AP1 | 1.24 | 0.62 | 2.45 | 0.5400 |  | 1.14 | 0.76 | 1.71 | 0.5300 |
| 209499_x_at | TNFSF13 | 1.18 | 0.69 | 2.01 | 0.5400 |  | 0.92 | 0.60 | 1.41 | 0.6900 |
| 210844_x_at | CTNNA1 | 0.86 | 0.54 | 1.37 | 0.5400 |  | 1.44 | 0.86 | 2.39 | 0.1600 |
| 218009_s_at | PRC1 | 1.23 | 0.63 | 2.41 | 0.5400 |  | 0.86 | 0.56 | 1.33 | 0.5000 |
| 201287_s_at | SDC1 | 1.19 | 0.65 | 2.17 | 0.5600 |  | 0.76 | 0.50 | 1.15 | 0.2000 |
| 204649_at | TROAP | 1.18 | 0.69 | 2.02 | 0.5600 |  | 1.23 | 0.75 | 2.00 | 0.4100 |
| 212589_at | RRAS2 | 0.87 | 0.54 | 1.39 | 0.5600 |  | 0.88 | 0.57 | 1.35 | 0.5600 |
| 219206_x_at | TMBIM4 | 0.84 | 0.46 | 1.52 | 0.5600 |  | 1.73 | 1.02 | 2.94 | 0.0410 |
| 204570_at | COX7A1 | 0.87 | 0.53 | 1.41 | 0.5700 |  | 1.18 | 0.77 | 1.81 | 0.4400 |
| 204306_s_at | CD151 | 1.20 | 0.64 | 2.24 | 0.5800 |  | 0.86 | 0.58 | 1.26 | 0.4400 |
| 213417_at | TBX2 | 1.20 | 0.64 | 2.24 | 0.5800 |  | 1.49 | 0.90 | 2.45 | 0.1200 |
| 201848_s_at | BNIP3 | 0.87 | 0.52 | 1.47 | 0.6000 |  | 0.92 | 0.61 | 1.38 | 0.6900 |
| 210507_s_at | AVIL | 0.87 | 0.52 | 1.46 | 0.6000 |  | 1.04 | 0.69 | 1.57 | 0.8400 |
| 212622_at | TMEM41B | 1.13 | 0.72 | 1.78 | 0.6000 |  | 0.84 | 0.53 | 1.33 | 0.4500 |
| 217798_at | CNOT2 | 1.14 | 0.70 | 1.87 | 0.6000 |  | 1.32 | 0.90 | 1.94 | 0.1600 |
| 200621_at | CSRP1 | 1.16 | 0.65 | 2.08 | 0.6200 |  | 1.14 | 0.79 | 1.64 | 0.4800 |
| 205559_s_at | PCSK5 | 1.17 | 0.63 | 2.20 | 0.6200 |  | 1.16 | 0.77 | 1.73 | 0.4800 |
| 221524_s_at | RRAGD | 1.19 | 0.60 | 2.35 | 0.6200 |  | 0.74 | 0.47 | 1.15 | 0.1800 |
| 212256_at | GALNT10 | 1.14 | 0.67 | 1.94 | 0.6300 |  | 0.74 | 0.47 | 1.17 | 0.2000 |
| 203562_at | FEZ1 | 0.88 | 0.51 | 1.51 | 0.6400 |  | 1.15 | 0.73 | 1.79 | 0.5500 |
| 205200_at | CLEC3B | 1.12 | 0.68 | 1.84 | 0.6400 |  | 1.13 | 0.75 | 1.70 | 0.5500 |
| 202729_s_at | LTBP1 | 0.87 | 0.47 | 1.61 | 0.6500 |  | 1.10 | 0.72 | 1.68 | 0.6500 |
| 209667_at | CES2 | 1.16 | 0.61 | 2.22 | 0.6500 |  | 0.75 | 0.48 | 1.17 | 0.2000 |
| 219004_s_at | C21orf45 | 0.92 | 0.64 | 1.32 | 0.6500 |  | 0.97 | 0.66 | 1.43 | 0.8900 |
| 218736_s_at | PALMD | 0.87 | 0.45 | 1.65 | 0.6600 |  | 1.60 | 1.01 | 2.54 | 0.0450 |
| 203071_at | SEMA3B | 0.87 | 0.47 | 1.64 | 0.6700 |  | 1.09 | 0.68 | 1.75 | 0.7100 |
| 203688_at | PKD2 | 0.89 | 0.52 | 1.53 | 0.6700 |  | 0.85 | 0.55 | 1.30 | 0.4500 |
| 206914_at | CRTAM | 1.17 | 0.57 | 2.42 | 0.6700 |  | 0.92 | 0.60 | 1.40 | 0.7000 |
| 209220_at | GPC3 | 1.17 | 0.57 | 2.38 | 0.6700 |  | 0.82 | 0.47 | 1.43 | 0.4800 |
| 219167_at | RASL12 | 1.15 | 0.61 | 2.16 | 0.6700 |  | 0.95 | 0.60 | 1.48 | 0.8000 |
| 201286_at | SDC1 | 1.13 | 0.63 | 2.03 | 0.6800 |  | 0.75 | 0.50 | 1.14 | 0.1700 |
| 205931_s_at | CREB5 | 0.89 | 0.50 | 1.56 | 0.6800 |  | 1.17 | 0.77 | 1.79 | 0.4700 |
| 209257_s_at | CSPG6 | 0.89 | 0.50 | 1.57 | 0.6800 |  | 1.02 | 0.66 | 1.56 | 0.9400 |
| 213316_at | KIAA1462 | 0.90 | 0.54 | 1.49 | 0.6800 |  | 1.02 | 0.66 | 1.56 | 0.9400 |
| 203017_s_at | SSX2IP | 0.90 | 0.53 | 1.53 | 0.6900 |  | 1.03 | 0.69 | 1.53 | 0.8900 |
| 204862_s_at | NME3 | 1.12 | 0.63 | 2.00 | 0.7000 |  | 0.99 | 0.63 | 1.54 | 0.9600 |
| 208703_s_at | APLP2 | 0.91 | 0.56 | 1.48 | 0.7000 |  | 0.80 | 0.56 | 1.16 | 0.2400 |
| 204821_at | BTN3A3 | 0.90 | 0.53 | 1.54 | 0.7100 |  | 0.73 | 0.47 | 1.11 | 0.1400 |
| 221519_at | FBXW4 | 1.12 | 0.62 | 2.00 | 0.7100 |  | 1.49 | 0.90 | 2.45 | 0.1200 |
| 200973_s_at | TSPAN3 | 1.10 | 0.65 | 1.88 | 0.7200 |  | 0.76 | 0.49 | 1.18 | 0.2200 |
| 201474_s_at | ITGA3 | 0.89 | 0.45 | 1.74 | 0.7300 |  | 0.66 | 0.39 | 1.11 | 0.1200 |
| 220351_at | CCRL1 | 0.91 | 0.54 | 1.53 | 0.7300 |  | 1.16 | 0.79 | 1.71 | 0.4400 |
| 204929_s_at | VAMP5 | 0.91 | 0.53 | 1.56 | 0.7400 |  | 0.66 | 0.40 | 1.07 | 0.0900 |
| 209264_s_at | TSPAN4 | 1.11 | 0.58 | 2.14 | 0.7500 |  | 0.85 | 0.58 | 1.26 | 0.4200 |
| 202177_at | GAS6 | 0.91 | 0.49 | 1.68 | 0.7600 |  | 1.05 | 0.73 | 1.51 | 0.7900 |
| 217287_s_at | TRPC6 | 0.92 | 0.52 | 1.62 | 0.7600 |  | 1.25 | 0.79 | 1.98 | 0.3400 |
| 215684_s_at | ASCC2 | 1.07 | 0.68 | 1.68 | 0.7700 |  | 0.88 | 0.54 | 1.44 | 0.6100 |
| 201061_s_at | STOM | 0.93 | 0.57 | 1.53 | 0.7800 |  | 0.93 | 0.59 | 1.44 | 0.7300 |
| 208777_s_at | PSMD11 | 1.07 | 0.67 | 1.72 | 0.7800 |  | 0.99 | 0.60 | 1.62 | 0.9600 |
| 209408_at | KIF2C | 1.07 | 0.63 | 1.82 | 0.7900 |  | 0.90 | 0.60 | 1.36 | 0.6200 |
| 212290_at | SLC7A1 | 1.07 | 0.67 | 1.71 | 0.7900 |  | 1.82 | 1.13 | 2.93 | 0.0140 |
| 38241_at | BTN3A3 | 0.93 | 0.56 | 1.55 | 0.7900 |  | 0.62 | 0.39 | 0.98 | 0.0420 |
| 209263_x_at | TSPAN4 | 0.91 | 0.46 | 1.82 | 0.8000 |  | 0.88 | 0.59 | 1.32 | 0.5300 |
| 205898_at | CX3CR1 | 1.06 | 0.62 | 1.82 | 0.8300 |  | 0.83 | 0.50 | 1.37 | 0.4700 |
| 211138_s_at | KMO | 1.06 | 0.61 | 1.85 | 0.8300 |  | 0.78 | 0.47 | 1.30 | 0.3400 |
| 211519_s_at | KIF2C | 0.94 | 0.54 | 1.64 | 0.8300 |  | 0.91 | 0.59 | 1.40 | 0.6600 |
| 211762_s_at | KPNA2 | 1.07 | 0.58 | 1.98 | 0.8300 |  | 0.85 | 0.54 | 1.34 | 0.4800 |
| 201884_at | CEACAM5 | 1.06 | 0.60 | 1.86 | 0.8400 |  | 0.79 | 0.46 | 1.36 | 0.4000 |
| 209785_s_at | PLA2G4C | 1.06 | 0.63 | 1.78 | 0.8400 |  | 0.84 | 0.54 | 1.32 | 0.4600 |
| 202071_at | SDC4 | 0.94 | 0.49 | 1.80 | 0.8500 |  | 0.62 | 0.40 | 0.97 | 0.0370 |
| 203571_s_at | C10orf116 | 0.94 | 0.53 | 1.70 | 0.8500 |  | 1.09 | 0.70 | 1.69 | 0.7100 |
| 203757_s_at | CEACAM6 | 0.95 | 0.56 | 1.63 | 0.8500 |  | 0.54 | 0.32 | 0.90 | 0.0200 |
| 212071_s_at | SPTBN1 | 0.95 | 0.54 | 1.67 | 0.8500 |  | 0.94 | 0.60 | 1.48 | 0.8000 |
| 202746_at | ITM2A | 0.94 | 0.51 | 1.76 | 0.8600 |  | 0.70 | 0.42 | 1.16 | 0.1700 |
| 203002_at | AMOTL2 | 0.95 | 0.53 | 1.69 | 0.8600 |  | 1.08 | 0.75 | 1.55 | 0.6700 |
| 204428_s_at | LCAT | 1.05 | 0.59 | 1.87 | 0.8600 |  | 0.73 | 0.46 | 1.15 | 0.1800 |
| 210325_at | CD1A | 0.95 | 0.51 | 1.76 | 0.8600 |  | 1.00 | 0.68 | 1.47 | 1.0000 |
| 219890_at | CLEC5A | 1.09 | 0.44 | 2.71 | 0.8600 |  | 0.92 | 0.59 | 1.44 | 0.7200 |
| 205347_s_at | TMSL8 | 0.97 | 0.63 | 1.49 | 0.8800 |  | 1.01 | 0.63 | 1.62 | 0.9700 |
| 211080_s_at | NEK2 | 0.96 | 0.57 | 1.63 | 0.8900 |  | 0.86 | 0.55 | 1.33 | 0.4900 |
| 211276_at | TCEAL2 | 1.04 | 0.57 | 1.90 | 0.9000 |  | 0.84 | 0.53 | 1.33 | 0.4500 |
| 201606_s_at | PWP1 | 0.98 | 0.60 | 1.60 | 0.9300 |  | 0.98 | 0.63 | 1.51 | 0.9200 |
| 201655_s_at | HSPG2 | 1.03 | 0.53 | 2.00 | 0.9300 |  | 1.26 | 0.81 | 1.96 | 0.3000 |
| 205713_s_at | COMP | 1.02 | 0.64 | 1.62 | 0.9300 |  | 0.92 | 0.60 | 1.42 | 0.7200 |
| 218349_s_at | ZWILCH | 1.02 | 0.61 | 1.71 | 0.9300 |  | 0.98 | 0.67 | 1.44 | 0.9200 |
| 202411_at | IFI27 | 1.03 | 0.51 | 2.07 | 0.9400 |  | 0.83 | 0.53 | 1.30 | 0.4200 |
| 201809_s_at | ENG | 1.02 | 0.59 | 1.75 | 0.9500 |  | 1.00 | 0.64 | 1.55 | 0.9900 |
| 206595_at | CST6 | 0.99 | 0.55 | 1.78 | 0.9600 |  | 0.83 | 0.51 | 1.36 | 0.4600 |
| 218686_s_at | RHBDF1 | 0.99 | 0.54 | 1.80 | 0.9700 |  | 0.89 | 0.58 | 1.36 | 0.5800 |
| 205306_x_at | KMO | 0.99 | 0.55 | 1.78 | 0.9800 |  | 0.90 | 0.56 | 1.45 | 0.6600 |
| 205495_s_at | GNLY | 0.99 | 0.61 | 1.63 | 0.9800 |  | 0.69 | 0.39 | 1.24 | 0.2200 |
| 205498_at | GHR | 1.00 | 0.63 | 1.57 | 0.9800 |  | 0.65 | 0.31 | 1.34 | 0.2400 |
| 208873_s_at | C5orf18 | 1.00 | 0.62 | 1.61 | 0.9900 |  | 1.15 | 0.74 | 1.80 | 0.5300 |
| 213169_at | NA | 1.00 | 0.54 | 1.85 | 1.0000 |  | 1.29 | 0.81 | 2.05 | 0.2800 |
